# 2016 AAPM Spring Clinical Meeting ‐ Abstracts

**DOI:** 10.1120/jacmp.v17i3.6370

**Published:** 2016-05-08

SATURDAY, MARCH 5

Best Poster Competition Exhibit Hall

PO‐BPC‐Exhibit Hall‐01

## Failure to Detect and Interlock an Out‐Of‐Tolerance Photon Beam Symmetry by a Linear Accelerator Monitor Chamber

N Knutson,* N Nguyen, M Schmidt, M Price

*Rhode Island Hospital, Providence, RI, USA*

### Purpose

The purpose of this work is to describe a scenario where a linear accelerator failed to detect large changes in symmetry and output over the course of the treatment day due to target failure.

### Methods

During routine after‐hour patient‐specific quality assurance (QA), a ∼10% change in the 6 MV cross‐calibration factor was observed. Review of morning QA results indicated beam parameters were within tolerance. Repeating morning QA measurements indicated symmetry and output values substantially outside of tolerance. The linac continued to operate without interlock and further investigation was warranted using a variety of dosimeters to confirm beam characteristics.

### Results

Investigation using a calibrated 2D diode array demonstrated a large change in the 6 MV beam's radial symmetry of ∼10%. Additionally, measurement with ADCL‐calibrated ion chamber confirmed a ∼10% decrease in output since the completion of morning QA. The 10 MV beam was confirmed to be within tolerance, indicating a faulty 6 MV target. To confirm, the target's position was shifted slightly which resulted in the beam symmetry and output returning to within tolerance. Visual inspection of the removed target showed signs of degradation, confirming our suspicions. To function in this state without interlock, it is hypothesized that the deficit in charge from photons coming from the target at the monitor chamber was replaced by an equal charge coming from primary electrons now escaping the target. Thus, the asymmetry of the photon profile measured at depth did not correlate with the charge collected on each side of the radial monitor chamber which should have triggered an interlock.

### Conclusion

Large changes in symmetry and output during the treatment day can go undetected by the linear accelerator monitor chambers. This highlights the need for possible increased QA frequency or perhaps a redesign of the monitor chamber system.

PO‐BPC‐Exhibit Hall‐02

## Graphical User Interface for Digital Winston Lutz Test‐Based Localization of Linac Radiation Isocenter and IGRT Image Centers

W Du

*University of Texas MD Anderson Cancer Center, Houston, TX, USA*

### Purpose

Digital Winston‐Lutz (DWL) tests have been increasingly used in radiation therapy quality assurance (QA) to replace the traditional film‐based WL tests. The radiation isocenter of a linear accelerator can be determined precisely with DWL using the digital images of a ball bearing (BB) phantom and square or circular radiation fields. The DWL tests have also been used to check the spatial accuracy for on‐board IGRT systems. There is a growing need of a software package for these DWL‐based QA procedures.

### Methods

A MATLAB graphics user interface (GUI) was developed to process the DWL images acquired on multiple linacs equipped with a variety of IGRT systems. The centers of the BB and the radiation fields were determined with subpixel accuracy using Hough transform algorithms. The radiation isocenter was located with respect to the center of the BB. The IGRT image centers were also determined relative to the center of the BB. Finally, the congruence of the image centers and the radiation isocenter was calculated for each IGRT system including OBI, EPID, CBCT and ExacTrac.

### Results

The GUI computed the locations of the radiation isocenter and the image centers in under 1 min. Repeated DWL tests showed reproducible results of the congruence of IGRT image centers and the radiation isocenter. The reproducibility of the results indicated (1) the mechanical stability of the linacs, (2) the independence of the image centers vs. radiation isocenter congruence on the phantom position, and (3) the accuracy of data analysis. The GUI was able to process images from a Varian 2100 linac and an Elekta Versa linac.

### Conclusion

The DWL‐based GUI provided a platform on which the radiation isocenter of a linac was located quickly and accurately, and the IGRT image centers were checked against the radiation isocenter.

PO‐BPC‐Exhibit Hall‐03

## Objective Assessment of Spine Phantom Using Various 3D Printers for Patient‐Specific QA in SBRT: a Feasibility Study

S Lee,^1,2*^ T Suh,^1,2^ M Kim,^1,2^ M Lee,^1,2^ S Park,^1,2^ J Song,^1,2^ J Sohn^3^

*Dept. of Biomedical Engineering,^1^ College of Medicine, The Catholic University of Korea, Seoul, Research Institute of Biomedical Engineering,^2^ The Catholic University of Korea, Seoul, Case Western University,^3^ Cleveland, OH, USA*

### Purpose

Radiation therapy oncology group (RTOG) 0631 suggest different planning method in spine stereotactic body radiation therapy (SBRT) according to location of cancer owing to its distinct shape. The purpose of this study is to verify dosimetric accuracy of delivered dose in spine SBRT as highly precise radiotherapy depending on cancer position using dedicated spine phantom based on 3D printer.

### Methods

The phantom was designed to evaluate delivered dose by three different cancer positions located in vertebral body, encircling the spinal cord, and the posterior spinous process supplied by RTOG 0631. Furthermore, the phantom was made to enable film to insert between two slabs of the phantom to verify the dosimetric effects and accuracy of dose distributions. For 3D printing, the phantom modeling was performed by various software applications such as SEG3D, ImageVis3D, Meshmixer, Solid Works, and CAD, based on spine CT images. There are many kinds of 3D printing methods depending on materials, printing time, or printing cost. Developed phantom can verify two classes according to printing Method: (i) direct light projection (DLP) method, (ii) polyjet method. These printing methods are using same material as acrylic polymer, the density of which is 1.39 g/ml similar with the density of spine.

### Results

The Hounsfield unit (HU) value of the acquired CT images was different in accordance with 3D printing method despite same material. Average HU values of DLP and polyjet method were 148.97 and 109.54 in same region of interest (ROI). The HU value of former method was similar with that of patient spine CT, which is 163.07.

### Conclusion

The developed phantom especially using DLP method can be utilized as spine SBRT dosimetry research. Our study was able to confirm that the phantom was indeed similar with HU value of human spine as well as its shape.

PO‐BPC‐Exhibit Hall‐04

## SAVI Quality Assurance Using Failure Modes and Risk Analysis Methodology

H Norris,^*^ V Gutti, G Palaniswaamy

*Baylor Scott & White Health Vasicek Cancer Center, Temple, TX, USA*

### Purpose

Standardized work and processes for radiation therapy are instrumental in providing high‐quality patient care in an efficient manner. We are developing standardized procedures for brachytherapy, stereotactic, and IMRT treatments based on process mapping, GEMBA, and FMEA. In this abstract, we present our work on workflow standardization for brachytherapy SAVI program for breast treatments. Scope of our work is directed towards reduction of human error and prevention of failures; and establishment of standardized procedures, personnel training, and high‐quality patient care.

### Methods

The entire process for SAVI treatment at our clinic was mapped by defining the 27 tasks in total performed by physicians, nurses, therapists, and physicists. Descriptions of tasks and roles were outlined. Performing GEMBA for each task, we identified “pain points” or potential modes of failure. We then compared the observed procedures with written departmental procedures and checklists, updating and/or establishing new procedures and policies. We are conducting FMEA with a quantitative RPN ranking to create and evaluate process controls to prevent potential failure modes from reaching the patient. As part of continuing process evaluation, we will track failure modes (e.g., replan or cancelled treatment) and compare rates with those prior to establishing this standardized work and processes.

### Results

A clear SAVI treatment process map was established with assigned task owners. For each task, a standard work procedure and relevant checklists were established. Personnel involved in SAVI treatment processes (simulation process, treatment planning, and delivery) are trained with new established workflow and procedures geared towards high‐quality patient care.

### Conclusions

Standard work procedures can lead to efficient, improved quality and performance in the radiation oncology setting. Established standard workflow at our clinic keeps members of the SAVI team clear as to their roles and processes, help reduce potential modes of failure and, in turn, improve quality of patient care.

PO‐BPC‐Exhibit Hall‐05

## Generating Nodule‐Like Object Functions for CAD Performance Evaluation in Lung Cancer Screening: Feasibility Study

A Narita,^*^ M Ohkubo, S Wada

*Niigata University, Niigata, Japan*

### Purpose

To evaluate the performance of computer‐aided detection (CAD) in lung cancer computed tomography (CT) screening, we propose a method of using ‘nodule‐like object functions’. Nodule‐like object functions are calculated from pulmonary nodules in clinical images by deconvolution analysis based on the spatial resolution of a CT scanner. They are applicable for any screening sites to generate computer‐simulated (virtual) nodules by convolution based on the spatial resolution of the site's scanner, allowing site‐specific realistic nodule generation. Such virtual nodules will be useful in evaluating site‐specific CAD performance. To demonstrate the feasibility of the methodology, we performed a pilot study using spherical objects in a phantom.

### Methods

We used a CT test phantom including spherical objects with diameters of 3, 5, 7, and 10 mm, and a contrast between sphere and background density of 674 Hounsfield units (HU). The sphere images obtained by a scanner (scanner‐1) were deconvolved with the point spread function (PSF) and slice sensitivity profile (SSP) measured for scanner‐1; obtained images were referred to as ‘nodule‐like object functions’. They were compared with ideal object functions. Next, by convolving the nodule‐like object functions with the PSF/SSP of a different scanner (scanner‐2), virtual nodules were generated, then compared with real images obtained by scanner‐2. The image differences were quantified by the root‐mean‐square error (RMSE).

### Results

The nodule‐like object functions generated from scanner‐1 images agreed well with the ideal object function, suggesting the validity of our deconvolution method. Virtual nodules generated from those functions were identical to the nodules in a real image obtained by scanner‐2; the RMSE values for 3‐, 5‐, 7‐, and 10‐mm diameter spheres were approximately 11.4, 13.7, 16.1, and 18.4 HU, respectively.

### Conclusion

The proposed method was demonstrated to be feasible, and is a potential method for the quality assurance of CAD.

PO‐BPC‐Exhibit Hall‐06

## Peripheral Dose Evaluation to the Thyroid and Contralateral Breast From Accuboost Treatments

M Talmadge,^*^ P Halvorsen, I Iftimia

*Lahey Clinic, Burlington, MA, USA*

### Purpose

To estimate the potential dose to the thyroid and contralateral breast for patients undergoing Boost, as well as accelerated partial breast irradiation (APBI), treatments using the Accuboost (Advanced Radiation Therapy, Tyngsboro, MA) surface brachytherapy system.

### Methods

Peripheral measurements were made using Optically Stimulated Luminescent Dosimeters (OSLD) (Landauer Inc., Glenwood, IL) with phantoms simulating patient anatomy under varying clinical set ups.

### Results

Recognizing that variability in both patient anatomy and treatment setup would have a significant effect on the dose to these peripheral structures, a series of measurements and analysis was performed to determine an approximate range of the potential cumulative dose to the thyroid and contralateral breast for a hypothetical patient undergoing either treatment regimen, taking into account the effects of the applicator type and size as well as the treatment separation distance. It was estimated that the potential dose to the thyroid ranged from 15 cGy to 36 cGy and from 44 cGy to 109 cGy for a full course of Boost and APBI treatments, respectively. For the contralateral breast, the dose was estimated to be from 38 cGy to 84 cGy and from 116 cGy to 257 cGy for Boost and APBI treatments, respectively.

### Conclusion

These results build upon previously published peripheral measurements made using the Accuboost system,^(1)^ establishing an estimated range for the tissues of interest taking into consideration the effects of applicator selection and treatment separation distance. Peripheral dose, particularly to the contralateral breast, can be an important consideration for both Boost and ABPI treatments.

1. Khanal SP, Ouhib Z, Benda RK, Leventouri T. Evaluation of surface dose outside the treatment area for five breast cancer irradiation modalities using thermo‐luminescent dosimeters. Int J Cancer Ther Oncol. 2015;3(1).

**PO‐BPC‐Exhibit Hall‐07**

## Impact of Iterative Metal Artifact Reduction Algorithm on Scatter Contouring in Radiation Therapy Treatment Planning

S Anderson,^*^ D Brinkmann

*Mayo Clinic, Rochester, MN, USA*

### Purpose

To investigate whether CT datasets reconstructed with an iterative metal artifact reduction (IMAR) algorithm can be used for radiation therapy planning without further corrections for scatter, thus eliminating the time‐intensive need to contour metal artifacts.

### Methods

We selected patient CT datasets with metal artifact in the beam path. We analyzed four cases with different sources of metal artifact: dental fillings, hip implants, spinal implants, and breast expanders. For each patient, a plan was first calculated on the original dataset, with scatter contoured and reassigned to an HU of 0. The plan, with same field geometry and MU, was then calculated on the IMAR‐corrected images, without any contouring of artifacts. The dose statistics for target volumes and organs at risk were compared for the original and IMAR‐corrected plans.

### Results

Dose statistics for the planning structures agreed within 1.5 Gy and 2.2 cc, the largest differences being for small volumes on the order of a few cubic centimeters. The statistics for the IMAR‐corrected images had higher values, in general, than the uncorrected images. A qualitative comparison of the dose distributions indicated that there were no major differences. A patient dataset without metal artifact was reconstructed with and without IMAR, and a plan calculated on both datasets resulted in identical dose statistics.

### Conclusion

Plans calculated on the IMAR‐corrected images resulted in similar dose statistics to those obtained under the current clinical practice of contouring metal artifact and reassigning HU values. The largest differences were for small volumes, where small absolute changes Result in large relative differences. The fact that the dose statistics for the organs at risk with IMAR were in general higher than the current clinical practice lessens the risk of overdosing a critical structure using IMAR. The outlook for using IMAR to avoid the time‐consuming process of contouring metal artifact is promising.

PO‐BPC‐Exhibit Hall‐08

## Ion Chamber Internal Temperature Measurements and Simulations for Thermal Equilibration Times After Ambient Temperature Changes

D Saenz,^*^ N Kirby, A Gutierrez

*University of Texas HSC SA, San Antonio, TX, USA*

### Purpose

Temperature and pressure corrections are necessary to account for varying air mass in the sensitive volume of a vented ionization chamber (IC). Locations used to measure temperature (in room, in phantom) may not accurately represent true IC air temperature, especially with a temperature change from storage to irradiation environment. The purpose of the study was to characterize the IC air temperature dependence upon changes in ambient temperature and understand where to take temperature measurements.

### Methods

Thermal conduction properties were investigated by modifying a PTW 0.3cc 31013 Semi‐flex IC with a thermocouple replacing the central electrode. The IC temperature was recorded in three phantom geometries characteristic of common output measurements. The phantoms were kept at $∼15^{\circ }C$ before measurement in a treatment vault ($∼21^{\circ }C$). The chamber was at the vault prior to measurement. Finally, simulations were conducted to simulate the thermal conductivity properties of other common ICs.

### Results

Two thermal equilibria were recorded on different time scales. The chamber temperature initially dropped to that of the cool phantom in the first equilibrium but increased as the phantom itself equilibrated with room temperature. In a $25.5\times 25.5\times 23.4\;{\text{cm}}^{3}$ cube phantom, IC equilibration with the phantom required 3 min, while final equilibrium with the room required >24 hrs. In a 7.5 cm slab phantom, 2 min were required for a chamber at dmax to reach within 0.5° of the minimum temperature. In this geometry, the subsequent increase in temperature was observed over 2 hrs as the phantom reached room temperature. Over 3 hrs were required for final equilibration with a 2 cm slab phantom.

### Conclusion

In‐phantom temperature recording was far more accurate than measurement in the air. Wait times of 3 and 2 mins are needed for a cube and 7.5 cm slab phantom, respectively, to achieve 0.2% dosimetric accuracy.

PO‐BPC‐Exhibit Hall‐09

## Absolute Film Dosimetry for Stereotactic Radiosurgery and Stereotactic Body Radiotherapy Quality Assurance Using Gafchromic EBT3 Films

S Lu,^*^ N Wen, J Kim, Y Qin, Y Huang, B Zhao, I Chetty

*Henry Ford Health System, Detroit, MI, USA*

### Purpose

To evaluate the absolute dosimetric uncertainties of EBT3 films coupled with a flat‐bed document scanner for IMRT‐ and VMAT‐based SRS/SBRT quality assurance.

### Methods

In this study, 93 SRS/SBRT patient‐specific quality assurances were performed with Gafchromic EBT3 films and an Epson Expression 10000XL flat‐bed scanner. An integrated film dosimetry protocol was established with in‐house software to streamline a nine dose pattern delivery for dose calibration, calibration curve fitting, dose mapping from multiple color channels, and profile/gamma analysis. The acrylic phantom consisting of two 5 cm slabs (30 cm×30 cm) was used for patient QA. The phantom was set up to measure the dose plane in the coronal direction for all the cases except spinal tumors, where the dose plane was measured axially to capture dose drop off from the vertebral body to the cord. Point dose was measured in the high‐dose region within the target using a PTW pin point ion chamber. 3%/1 mm gamma passing rate was used for film analysis. A one‐way ANOVA was used to evaluate if any of the variables (volume size, treatment site, treatment delivery technique) had a statistically significant effect on the gamma index.

### Results

The percentage of points passing the 3%/1 mm gamma criteria, averaged over all tests was 94.5±5.7, with a corresponding 95% CLs between 93.4 and 95.8. No variables (volume size, treatment site, treatment delivery technique) were found to have a statistically significant impact on the gamma index based on the one‐way ANOVA test.

### Conclusion

Our film dosimetry protocol can offer a highly efficient solution for absolute dose commissioning and routine SRS/SBRT PSQA.

The work was supported by a research scholar grant, RSG‐15‐137‐01‐CCE from the American Cancer Society.

PO‐BPC‐Exhibit Hall‐11

## Determination of Linear Mapping Factors to Convert Corrected EPID Pixel Values for Dynamic Plan QA with Portal Dosimetry and MOSAIQ

M Schmidt,^*^ N Knutson, F Liu, M Price

*Rhode Island Hospital, Providence, RI, USA*

### Purpose

This works explains the method by which the electronic portal imaging device (EPID) on a Varian Medical Systems treatment machine may be used for fluence‐based patient‐specific IMRT quality assurance while utilizing MOSAIQ record and verify system.

### Methods

When an integrated image is transferred from the Varian treatment console to the MOSAIQ R&V, some of the DICOM tags essential for portal dosimetry are modified or removed. Two of those tags, R‐intercept and R‐slope, contain the mapping from relative pixel values to absolute dosimetry units calibrated upon commissioning of the amorphous silicon (aSi) panel. This work utilizes fields of various MU (50 MU–200 MU) and field size (5 cm×5 cm−20 cm×20 cm) to define a linear relationship between the slope and intercept values with the MU of the delivered field and introduces a simple tool to restore the R&V images to an acceptable format for analysis. These fields were extracted with and without the use of the R&V system and compared using the portal dosimetry application after making the necessary alterations to the R&V DICOM images.

### Results

These images were compared using a gamma analysis (1%/1 mm) and dose difference criteria with a region of interest selection of the field size with an additional 1 cm margin. All images were able to achieve a passing pixel percentage within their ROI of greater than 93%, with only one image not gaining 100% pass rate. The average dose difference for all images relative to their maximum value was 0.26%.

### Conclusion

The determination of pixel to calibrated unit mapping derived from a linear mapping relationship with the MU delivered proves effective in reinstating the absolute dosimetry of the integrated images imported into MOSAIQ.

PO‐BPC‐Exhibit Hall‐11

## A Sensitivity Dosimetric Study of Setup Uncertainties During Machine Commission and Annual QA

V Nguyen,^1*^ B Wang,^1^ C Shi^2^

*University Louisville, Louisville, KY, Saint Vincent Medical Center,^2^ Bridgeport, CT, USA*

### Purpose

To investigate three common potential setup uncertainties during linac commission & annual QA and evaluate how these setup uncertainties propagate into patient‐specific QA results using gamma index analysis.

### Methods

Three uncertainty scenarios were purposely introduced for gantry position tilts from 0°‐3° (scenario 1), SAD changes from 100‐103 cm (scenario 2), and isocenter position shifts from 0‐4 mm (scenario 3). Data were simulated using a $60\times 60\times 60\;{\text{cm}}^{3}$ solid water cube created in Varian Eclipse TPS (v.11) to replicate a 3D water tank. For each scenario, beam data profiles (crossline and diagonal) and percent depth dose were simulated individually at different field sizes and depths for three energies: 6 MV, 6MV‐FFF and 10MV‐FFF. A gamma analysis was used to evaluate the results using 1%/1 mm criteria.

### Results

Both PDDs and profiles showed larger deviations for increasing setup uncertainties as expected, while different patterns as a function of depth among the three scenarios. For example in scenario 1, a ≥90% gamma passing rate and ≤1 dose difference were observed for gantry tilted up to 2°. For 3° titled, gamma dropped below 90% at depth of ≥20 cm for 6MV/6FFF and depth of ≤ 12 cm for 10MV‐FFF. This is reasonable since gantry rotations cause larger geometric variations at larger depths. However for Scenario 2, dosimetric uncertainties were larger at shallower depths. Gammas were ≤90 and dose differences were ≥1% for depth up to 20 cm for all energies. For scenario 3, a ≥90% gamma passing rate and ≤1 dose difference were seen on ≤ 4 mm isocenter shifted for all energies.

### Conclusion

This study validated AAPM TG‐142 recommendations on the mechanical and dosimetry uncertainties and provided quantitative analysis of maximum acceptance tolerances for linac annual QA and commission.

PO‐BPC‐Exhibit Hall‐13

## Electromagnetic Interference Artifacts in Digital Radiography Caused by Left Ventricular Assist Devices (LVAD)

Z Lu,^1^ K Little,^1*^ I Reiser,^1^ V Kagan,^2^ A Sanchez,^1^ H MacMahon^1^

*Department of Radiology,^1^ University of Chicago, Chicago, IL, Thoracic Surgery,^2^ The University of Chicago Medicine & Biological Sciences, Chicago, IL, USA*

### Purpose

To identify and characterize artifacts caused by left ventricular assist devices (LVAD) on digital detector radiography images, and to investigate approaches to reduce these artifacts.

### Methods

When a digital detector is used to acquire a radiograph of a patient with an implanted device that is not electromagnetically compatible with the detector, electromagnetic interference (EMI) artifacts can occur on the acquired images. This was brought to our attention on multiple patients with different models of LVADs. Although the artifacts have similar repetitive linear patterns, the appearance, extent, and severity of the artifact vary widely between LVAD models and between patients. We tested a HeartMate II device with an anthropomorphic lung phantom on two digital detectors (Canon 70C, GE FlashPad) to study how distance, LVAD rotation speed, and the usage of a grid affect EMI artifacts.

### Results

The EMI artifacts on images were caused by the LVAD device. This was confirmed by acquiring two images with the LVAD on and off. There was no artifact in the LVAD‐off image. The EMI artifacts were affected by the distance between the LVAD device and the detector. Increasing the distance reduced the severity. The EMI artifacts were also affected by the rotation speed of the LVAD, and the artifacts were barely visible at the lowest motor speed tested. Adding a spacer between the patient and the detector may mitigate the artifacts. In addition, at least one vendor has developed an EMI artifact reduction feature to be used during image acquisition if EMI is known to exist.

### Conclusion

With more digital radiography units replacing computed radiography units, it is important to understand the physical limitations of digital detectors and to be vigilant for electromagnetic interference artifacts that are unique to the image acquisition process of such digital detector radiography units.

This work was supported in part by the RSNA/AAPM Imaging Physics Residency Program Grant.

PO‐BPC‐Exhibit Hall‐14

## Effect of Grid Size on Optimization Using the MultiPlan Monte Carlo Algorithm

R Krauss,^*^ J Tubbs

*St. Francis Hospital, Memphis, TN, USA*

### Purpose

To determine the effect of optimization grid size on the full resolution calculation of lung plans using MultiPlan's fast Monte Carlo algorithm by comparing visual prescription lines and new conformity index.

### Methods

Three lung lesions were contoured with a 5 mm PTV expansion in the MultiPlan treatment planning system. A set of beams was generated for each PTV using the Ray‐Tracing algorithm and the plan saved. Using the saved beam set, a single optimization was performed for each PTV using MultiPlan's Monte Carlo algorithm with identical constraints on low (64×64), medium (128×128), and high (512×512 “native”) resolution. No further optimization or time or beam reduction was done to ensure each resolution was optimized under the same conditions. Each plan was prescribed to 54 Gy. The conformity was then visualized in three planes and calculated using Paddick's new conformity index. All optimized plans were then calculated on high resolution and represcribed as above. The conformity was again visualized in the three planes and conformity calculated.

### Results

Comparison of the conformity of each low, medium, and high resolution optimization to the conformity of the corresponding high resolution calculation showed significant improvement in agreement between the optimization and final calculation as the resolution increased. Each optimization produced plans with high conformity (nCI=1.1–1.2) but at lower resolutions, the high resolution calculation deviated significantly from the original optimization (low: nCI=1.4−1.6, medium: nCI=1.2−1.4). High resolution optimizations were accurately reflected in the final high resolution calculation, as expected (nCI=1.1–1.2).

### Conclusion

This work indicates that optimizations using lower resolutions of MultiPlan's fast Monte Carlo algorithm do not accurately reflect the dose distribution calculated at the highest resolution. While high resolution optimizations can take two to three times longer than at lower resolutions, the conformity of the final plan may offset the additional optimization time.

PO‐BPC‐Exhibit Hall‐15

## Dosimetric Effect of Liver Motion for Scattering Proton Beam Therapy: a Proton Liver 4D Treatment Planning Strategy Study

Q Huang,^*^ M Zhang, W Zou, N Yue

*Rutgers Cancer Institute of New Jersey, New Brunswick, NJ, USA*

### Purpose

Intrafraction respiratory motion in liver leads to variation between delivered and planned tumor coverage in proton therapy. In this study, we investigated strategies for designing liver proton plans with double scattering technique to minimize this variation.

### Methods

Liver cancer patients with different tumor center motion treated with proton were retrospectively analyzed for the study. Free‐breathing scans (FB), maximum intensity projection scans (MIP), time‐averaged scan (Ave), and exhale phase scans (CT50) were used to create proton plans. Real‐time dose distribution was simulated by calculating dose in each phase of 4D CT with same beam setting for according strategy. The delivered dose was determined by accumulating dose deposition of all phases using deformable image registration implemented by VelocityAl software. Delivered tumor coverage was defined as percentage volume of GTV receiving at least prescribed dose in delivered dose distribution while planned tumor coverage was defined as percentage volume of PTV receiving at least 95% prescribed dose in planned dose distribution. Tumor coverage was calculated to evaluate delivered and planned dose distribution for four planning strategies.

### Results

Dosimetric variation between delivered and planned dose for each strategy were observed. For the current patient we analyzed, delivered tumor coverage were 99.92%, 95.45%, 99.92%, and 95.08% for FB, MIP, AIP, and CT50 plans, respectively. Corresponding planned tumor coverage were 97.54%, 98.9%, 99.66%, and 98.51%. Maximum cord dose, maximum esophagus dose, and small bowel doses were comparable between the planned and delivered dose. The Result indicates proton plans designed based on time averaged scans is a good indicator for delivered dose distribution.

### Conclusion

A systemic approach has been developed for comparing delivered and planned dose distribution for proton plans created on different static CT scans. The Result indicates proton plans based on time averaged scan predicts tumor coverage better.

PO‐BPC‐Exhibit Hall‐16

## Evaluation of Modified Newton Method Used for Inverting Nonlinear Transformations

P Greer,^1^ J Hatton,^2^ B McCurdy,^3^ P Siciarz,^3*^ C Tang^4^

*Calvary Mater Newcastle,^1^ Waratah, NSW, Australia, Mater Hospital,^2^ Callaghan, NSW, Australia, CancerCare Manitoba,^3^ Winnipeg, MB, Canada, Sir Charles Gairdner Hospital,^4^ Nedlands, WA, USA*

### Purpose

The goal of this project was to evaluate the accuracy of Modified Newton Method (MNM) used for inverting forward nonlinear transformations obtained through CT‐CBCT deformable image registration.

### Methods

Forward transformations were acquired by CT‐CBCT deformable registration applying dense anatomical block matching (DABM) algorithm that was proved to be superior for CT‐CBCT registration compared to well‐known B‐spline and Demons algorithms. To the best of author knowledge it is the first study which attempts to investigate the performance of Modified Newton Method used for inverting forward transformations of DABM algorithm. Pretreatment CT images of five prostate patients undergoing IMRT were selected for this work. CBCT datasets were acquired in the middle of the radiotherapy treatment. Deformable registration for each algorithm was followed by the initial rigid alignment of considered images. Backward transformations were than obtained by MNM that inverted forward transformations. The accuracy of Modified Newton Method was assessed by the application of inverse consistency error (ICE). Detailed analysis consists of: ICE maps, normalized distributions of ICE over the patient's volume, mean value of ICE, as well as the correlation between ICE and Jacobian determinants of forward transformations.

### Results

Early results clearly indicate very promising performance of MNM for challenging task of inverting nonlinear transformations. The most frequently received values of ICE are below 0.005 mm for all five patients, with mean ICE staying in the range from 0.0034 mm to 0.0058 mm. Also, negative correlation was found between ICE and Jacobian determinant which means that the more the transformation is preserved, the smaller ICE is expected.

### Conclusion

Modified Newton Method allows to accurately calculate inverse transform for DABM algorithm in the case of CT‐CBCT deformable registration for patients with prostate cancer. Observed lack of large ICE values makes the method reliable for calculations of accumulated dose delivered to patients in adaptive radiation therapy applications.

Cancer Care Manitoba; University of Manitoba

PO‐BPC‐Exhibit Hall‐17

## Quantification the Dosimetric Impact of the Elekta IBEAM Evo Couchtop EP in Different Beam Energies

R Zhang,^1^ Y Gao,^2^ W Bai,^1*^ X Fan,^1^ Z Chi^1^

*The Fourth Hospital of Hebei Medical University,^1^ Shijiazhuang, Hebei, Hebei General Hospital,^2^ Shijiazhuang, Hebei, China*

### Purpose

To quantification the dosimetric impact of the Elekta couchtop EP in different beam energies and different depths were measured to evaluate the impact of treatment couch on surface dose distributions.

### Methods

Film measurements were performed with EBT3 film with an MLCi2‐shaped of a $10\times 10\;{\text{cm}}^{2}$ field on a Solid Water phantom surface. Measurements were performed at different depths (1 mm, 5 mm, 10 mm, 15 mm, 20 mm, and 25 mm) at an SSD=100 cm, irradiated with 400 MU. The absorbed doses of deliveries at gantry angles 180° and 0° were compared. Using the ImageJ software to extract the digitized films red channel intensity value, and the intensity value is adjusted to 1 MU corresponds to a dose of 1 cGy at the depth of dose maximum. By interpolation to get the percent depth‐dose curves with and without the couchtop at the gantry angle 180° and 0°.

### Results

The couchtop EP showed a significant change in surface dose from 184 cGy, 140 cGy, and 117 cGy to 383 cGy, 352 cGy, and 296 cGy with the introduction of the treatment couch into the 6 MV, 10 MV, and 18 MV beam at the phantom depth 1 mm, increased 109%, 151%, and 153%, respectively. The depth of maximum dose also decreased by almost 10 mm across all experimental setups for all the beam energies we have measured when the couchtop EP was introduced into the treatment beam.

### Conclusion

When the beam intersects the treatment couch surface the buildup effect occurred in a clinical setting. The couchtop EP can approximately double the surface dose, relative to the maximum delivered dose, on the skin of the patient which should be taking more notice. This buildup interference by the treatment couch is most prevalent for lower treatment energies.

PO‐BPC‐Exhibit Hall‐18

## Varian Surface Applicator Leakage Measurements

M Talmadge,^*^ P Halvorsen, I Iftimia

*Lahey Clinic, Burlington, MA, USA*

### Purpose

To experimentally quantify the magnitude and extent of leakage radiation surrounding the Varian Surface Applicators (Varian Medical Systems, Palo Alto, CA) when loaded with an Ir‐192 high‐dose‐rate source.

### Methods

Peripheral measurements were made using Gafchromic EBT3 film (Ashland Inc., Wayne, NJ) as well as Optically Stimulated Luminescent Dosimeters (OSLD) (Landauer Inc., Glenwood, IL) and compared to the predictions of the Varian Acuros BV treatment planning system (Varian Medical Systems). The OSLD system was calibrated using 6 MV X‐rays, whereas the film‐based measurements were normalized linearly based the film's response at a prescription depth of 4 mm as the absolute dose at this point was validated during commissioning. All measurements were made using a single source dwell position at 15 mm from the distal end of the source path, consistent with clinical use.

### Results

Given the inherent uncertainties presented by large dose gradients around the applicator, the measured results are in reasonably good agreement with those predicted by the treatment planning system; however, the film measurements indicate that the spatial extent of the leakage radiation field around the applicators may be greater than that predicted by the treatment planning system.

### Conclusion

There is nonnegligible leakage radiation surrounding the proximal portion of the Varian Surface Applicators that should be taken into account during clinical use when evaluating potential dose to nearby tissues that may be in the proximal direction along the source path relative to the treatment surface.

PO‐BPC‐Exhibit Hall‐19

## Fade and Warm‐Up of MOSFET Dosimeters

D Plenkovich

*Memorial Hospital of Sweetwater County, Rock Springs, WY, USA*

### Purpose

It is generally believed that the MOSFET dosimeters have fade, or that their response changes with time. The fade component of the MOSFET dosimeter's response is supposed to be <3%. In this presentation, it will be demonstrated that in the mobileMOSFET system (Best Medical Canada) fade can be almost completely eliminated with a proper warm‐up.

### Methods

Five standard sensitivity, single MOSFET dosimeters were used in this study. They were taped on the Calibration Jig, which was placed on 7 cm of virtual water slabs for backscatter. The MOSFET dosimeters were covered with 1.5 cm thick bolus. The SSD to surface of bolus was 100 cm, and the field size was 10 cm×10 cm. The MOSFET dosimeters were irradiated with 100 MU and the 6 MV photon beam, the output of which has been calibrated and adjusted. The sequential reading feature in the mobileMOSFET software enables reading the dose data periodically or in predetermined intervals. The incremental dose to the MOSFET dosimeters was read in 60 s intervals.

### Results

The fade component of the radiation dose was significant in the first 3 hrs of measurement. 3.5 hrs after the irradiation of dosimeters, the measured dose leveled off very close to the expected value of 100 cGy. No fade effect could be seen.

### Conclusion

The MOSFET dosimeters must be connected to the reader, which should have its power turned on. About 3.5 hrs after the irradiation, the mobileMOSFET system reads the correct dose with almost no fade effect. This is the optimal time for calibration of the MOSFET dosimeters and for performing patient or beam output measurements.

Best Medical Canada has provided MOSFET dosimeters and buildup caps for this project.

PO‐BPC‐Exhibit Hall‐20

## Application of Taguchi Methodology for Optimizing Parameters of Cone‐Beam CT for Optimal Image Quality

S alani,1^*^ A Schlocker,^2^ N Honig,^3^ G Elikhis,^3^ M Levita^3^

*Tel Aviv Sourasky Medical Center,^1^ Tel Aviv, Tel Aviv Medical Center,^2^ Modiin, Tel Aviv Medical Center,^3^ Tel Aviv, Israel*

### Purpose

Taguchi Method is a statistical approach to optimizing multiple process parameters to determine a combination resulting in the best output. This study utilizes the Taguchi Method to evaluate the image quality parameters of Varian OBI v2 CBCT for adaptive radiotherapy image sets. The CATPHAN 600 CT phantom, which includes several modules for measuring image quality indices for imaging protocols' various parameters, was utilized.

### Methods

Taguchi developed a unique mathematical design of orthogonal arrays to study an entire parameter space with few experiments. The experimental results are transformed into what Taguchi referred to as signal‐to‐noise ratio (t‐S/N) that is a measure of quality characteristics deviating from or nearing to a desired value. Analyses of variance (ANOVA) were employed to study the performance characteristics of the CBCT image quality. In this analysis, three factors of the acquisition protocol were considered: (a) CT slice thickness; (b) image reconstruction filter types; (c) matrix size. Respectively, a suitable orthogonal array was selected (3×3×3) to perform the experiments. After conducting the experiments the image quality parameters: pixel value stability, noise, contrast‐to‐noise ratio, and uniformity were measured, and t‐S/N for each parameter was calculated.

### Results

The quantified t‐S/N values analyzed in JMP software. The optimal combination of three factors was determined to be comprised of (a) 1 mm CT slice thickness; (b) “sharp” filter; (c) 512×512 matrix. The dominant factors influencing image quality are (b) filter type, and the cross interaction between the filter type and the CT slice thickness (aXb). Matrix size (c) plays a relatively minor role since it was incapable of yielding a 95% confidence level in the ANOVA test.

### Conclusion

The Taguchi Method provides simple, systematic, and efficient methodology for optimizing the process parameters for evaluating image quality for CBCT study sets and may allow us to improve adaptive radiotherapy as it matures.

PO‐BPC‐Exhibit Hall‐21

## Evaluation of Single Field Uniform Dose (SFUD) Proton Pencil Beam Scanning (PBS) Planning Strategy for Lung Tumor

G Liu,^1*^ X Li,^1^ D Yan,^1^ Quan,^2^ X Ding^1^

*Beaumont Health System,^1^ Royal Oak, MI, USA, Wuhan University,^2^ Wuhan, Hu Bei, China*

### Purpose

We quantitatively evaluate the actual dosimetric outcome based on four different SFUD PBS planning strategies.

### Methods

A virtual lung patient 4D CT images were generated by inserting a sphere with diameter of 3 cm representing a rigid motion target (GTV) to the right lung of a patient 4D CT. The target motion is set in superior–inferior(SI) direction from ‐5 mm to 5 mm with step size of 1 mm to simulate a rigid tumor motion. CT‐average, maximum intensity project (MIP) were generated. Four proton SFUD planning strategies were evaluated based on: (1) MIP‐CT; (2) CT_average with ITV override to muscle tissue (CTavg_Muscle); (3) CT average with ITV override to tumor density (CTavg_Tumor); (4) CT_average without any override density (CTavg_only). Dose distributions were recalculated on each individual phase and accumulated together to assess the ‘actual’ treatment. To estimate the impact of proton range uncertainties, ±3.5% CT calibration curve was applied to the 4D CT phase images. Dose‐volume histograms (DVHs) of GTV and Heart were analyzed.

### Results

Comparing the dose from initial plan with the dose accumulation: The ‘actual’ accumulated GTV D98 was 57.68 Gy; 53.48 Gy; 59.73 Gy; 60.4 Gy; Heart dose D1 increases from 1.88 Gy to 8.07 Gy; from 2.69 Gy to 2.96 Gy; from 3.74 Gy to 5.98 Gy; from 4.38 Gy to 7.82 Gy; In the presence of proton range uncertainties of ±3.5%, CTavg_tumor based plan's accumulated GTV D98 degraded to 57.99 Gy (+3.5%) 59.38 Gy (−3.5%), CTavg_Muscle based plan's accumulated GTV D98 degraged to 59.37Gy (+3.5%) 59.37 Gy (−3.5%).

### Conclusion

CTavg_Tumor and CTavg_Muscle provide the most robust GTV coverage. However, clinicians need to be careful about the dose to OARs at distal end of beam directions because the proton might stop further than the initial plans especially if plan is based on CTavg_Muscle. The study also indicates that the current SFUD PBS planning strategy might not be sufficient to compensate the CT calibration uncertainty.

PO‐BPC‐Exhibit Hall‐22

## Calibration and Comparison of Dosimetry Systems Used in the Therapeutic Soft X‐Ray Energy Range

S Souri,^*^ X Qian, Y Chen, A Jamshidi

*North Shore Long Island Jewish Health System, New Hyde Park, NY, USA*

### Purpose

The main objective of this study is to compare and evaluate different dosimetry systems which can be used as *in vivo* dosimetry for intraoperative radiotherapy in therapeutic soft X‐ray energy range.

### Methods

Optically stimulated luminescent dosimeter (OSLD) nanoDots and radiochromic‐EBT2 films evaluated in soft X‐ray energy produced by a Zeiss‐Intrabeam 50 kV radiotherapy system using 4 cm surface applicator. Solid water with different thicknesses used to simulate different depths. NanoDots irradiated in depths of 3 mm, 10 mm, and 20 mm while EBT2 films at surface and 20 mm depth. The responses of the nanoDots were measured by a LandauermicroSTAR‐ii reader system. For film dosimetry, an Epson scanner and RIT software used.

### Results

The nanoDot response in soft X‐ray energy was observed as big as a factor of 4 compared to that for 6 MV photons. In contrast, the EBT2 film OD in 50 kV drops a factor of 3 compared to that in 6 MV and is less than 0.05 below 50 cGy. In order to achieve a calibration accuracy of 3%, segmented linear calibration curves were used. The calibration curves yield nearly identical results for nanoDots at depths of 10 and 20 mm, while about 15% less at 3 mm depth, suggesting that the nanoDot sensitivity increase with increased X‐ray energy and reach a plateau for harder X‐ray beyond 1 cm water‐equivalent depth for 50 kVp X‐rays. However, for EBT2 films, OD at 2 cm depth increases about 25% with respective to that at surface, demonstrating slightly larger energy dependence than nanoDots.

### Conclusion

The nanoDot OSLD is a better system for use in soft X‐ray energy with greater sensitivity and less energy dependence compared to EBT2 films. Caution should be exercised in choosing an appropriate set of calibration when X‐ray beam hardening effect has to be taken into consideration. More detailed energy dependent effect for the nanoDots is under investigation.

PO‐BPC‐Exhibit Hall‐23

## Estimating Peak Skin Dose in the Cath Lab Using Gafchromic Film

A Scott,^*^ Y Zhou, J Nute, C Lee, J Allahverdian

*Cedars‐Sinai Medical Center, Los Angeles, CA, USA*

### Purpose

The FDA requires fluoroscopy units manufactured after 2006 to provide the displayed air kerma (DAK) measured at the Interventional Reference Point (IRP), which is 15 cm from the isocenter towards the tube. However, from a radiation safety perspective, the quantity of interest is the peak skin dose (PSD) as erythema may occur at a PSD threshold of 2 Gy. We used Gafchromic film to investigate a correlation between DAK and PSD in the catheterization lab.

### Methods

In order to relate dose to Gafchromic film optical density (OD), a batch of Gafchromic film was calibrated by exposing pieces to a range of AK levels (0.25 Gy to 10.0 Gy) using DSA mode in a GE cath lab while measuring the AK using a Radcal ion chamber. The film OD was measured by scanning the film with an Epson 10000XL, splitting the image into RGB channels, and measuring the red‐channel pixel values using ImageJ. A saturation‐growth model was fit to the distribution of pixel values vs. AK to provide an OD‐to‐dose calibration. To measure PSD, a sheet of film was placed on the patient table during a cardiac procedure and collected along with the accumulated DAK. Afterwards, the peak OD of the film was measured and the PSD was calculated using the calibration.

### Results

The PSD and uncertainties for 12 procedures were compared to accompanying DAK records and the ratio calculated. The average PSD calculation uncertainty was 6%. With the exception of one outlier, the average ratio of measured‐to‐displayed air kerma was 0.36±0.08 with a range of 0.28 to 0.49.

### Conclusion

We found a correlation between DAK and PSD in the procedures studied and can make an improved estimate of PSD compared to using beam time. This will improve the accuracy of radiation dose warnings for high‐dose cath lab procedures.

PO‐BPC‐Exhibit Hall‐24

## Setup Uncertainties in Locally Advanced Breast Cancer Patients Undergoing Regional Nodal Irradiation

Y Zhang,^*^ D Brinkmann, R Mutter, S Park, E Yan, D Pafundi, N Remmes

*Mayo Clinic, Rochester, MN, USA*

### Purpose

To evaluate the setup uncertainties of the regional nodal stations using on‐board kV imaging (OBI) when compared with the gold standard cone‐beam CT (CBCT).

### Methods

20 consecutive patients undergoing postmastectomy (n=14) or postlumpectomy (n=6) radiotherapy who required regional nodal irradiation were enrolled. The clinical target volume (CTV) included the breast/chestwall and regional lymph nodes (axillary, infraclavicular, supraclavicular, and internal mammary nodal regions) and was treated using either free breathing (n=9) or breath hold (n=11) technique. Daily patient setup was based on OBI matching to the ipsilateral anterior ribs and sternum. In order to encompass the entire CTV (20‐28 cm in the S/I direction), a multiscan CBCT, consisting of a superior and an inferior scan, were acquired at the treatment position and stitched offline for assessment of setup reproducibility. Offline matching to the breast/chest wall CTV or the regional node CTV was performed on the stitched volume and setup uncertainties were represented by the shift differences between the CTV matches and the treated bony anatomy OBI match.

### Results

A total of 168 CBCT sets were available for the analysis. Setup uncertainties (mean± SD) on the regional node CTV were −0.2 mm×2.2 mm, −0.3 mm×4.0 mm, and 0.3 mm±2.3mm in the L/R, A/P and S/I directions, respectively. Setup uncertainties on the breast/chestwall CTV were 0.4 mm±2.6mm, 0.5 mm±3.0mm, and −0.7 mm×3.6 mm in each direction, respectively. Setup uncertainties were not significantly different for patients treated with breath hold compared to free breathing (p>0.1). Similarly, there was no significant difference in setup uncertainties between patients with intact/reconstructed breasts compared to nonreconstructed chest walls (p>0.1).

### Conclusion

OBI matching to chest wall bony landmarks provides an acceptable localization surrogate for both the regional node CTV and the breast/chestwall CTV in lieu of daily CBCT. Daily OBI instead of CBCT also reduces radiation dose to the patient.

Professional Symposium ‐ SAM Grand Ballroom D *Ethics & Professionalism*

SA‐A‐BRD‐00

Ethics & Professionalism

Moderator: Michael Howard

*Parkridge Medical Ctr, Chattanooga, TN, USA*

SA‐A‐BRD‐01

Ethics: An Historical Perspective

P. Dunscombe

*The University of Calgary*

SA‐A‐BRD‐02

Ethical Issues in Medical Physics Education

N. Bahar

*Landauer Medical Physics*

SA‐A‐BRD‐03

Ethical Issues in Medical Physics Business

C. Serago

*Mayo Clinic*

SA‐A‐BRD‐04

Ethical Issues in Medical Physics Research

N. Ozturk

*The University of Chicago*

Ethical Issues in Medical Physics Business

Ethics and professionalism underpin everything the medical physicist does. Unfortunately these topics receive little prominence in education, training and particularly continuing professional development due in part, at least, to the relative paucity of succinct and accessible educational materials geared to the medical physicist. This SAM workshop will start with an overview of some of the more widely recognized ethical viewpoints: duty ethics, consequentialist ethics, and virtue ethics. The remainder of the workshop will be configured around the AAPM's Code of Ethics. The four major sections in the Guidelines in the Code of Ethics will be reviewed: Professional Conduct, Research Ethics, Education Ethics, and Business Ethics. The presentation of these topics will be informed by the results of the recent AAPM survey on ethics and professionalism. It is intended to combine the appeal of a SAM session with the interactive learning that takes place in a small group format. Participants will divide themselves into informal group of 5‐10 and discuss each SAM question for 5 min or so before individually using their clickers to respond to the question.

### Learning Objectives:

1. To appreciate the differences between the major schools of ethical thought.
2. To gain an overview of the content of the AAPM Code of Ethics.
3. To develop approaches, through discussion with colleagues, to recognizing and responding to ethical issues in our professional lives.

Young Investigator Symposium Grand Ballroom D

SA‐B‐BRD‐01

## A Novel Energy‐Dependent Subtraction Method for Cardiac Imaging: Signal and Noise Analysis

C Burton,^*^ I Cunningham

*Western University, London, ON, Canada*

### Purpose

Cardiovascular diseases (CVD) are currently the leading cause of death worldwide and X‐ray angiography is used for 80% of all cases. Digital subtraction angiography (DSA) is a technique that is widely used to enhance the visibility of vasculature obscured by overlying bone and lung fields by subtracting a mask and contrast image. However, DSA is generally unsuccessful for imaging the heart, due to the motion that occurs during the several seconds between mask and contrasted images that cause artifacts and can render a study nondiagnostic. We are proposing energy subtraction angiography (ESA) as a method of bringing the benefits of DSA to cardiac imaging without motion artifacts. This method was suggested in the 1970s and it was concluded at the time that image quality with ESA could not compete with that of DSA, and the approach was abandoned. However, our work has shown that conclusion was based on limitations of early technology that may be no longer relevant.

### Methods

We developed a theoretical model for iodine signal and noise to form a metric of comparison of iodine SNR between ESA and DSA independent of technology and validated it with experiment. Read‐noise and scatter were incorporated into our theoretical model.

### Results

The theoretical model showed that, in principle, ESA can produce images that are as good as DSA, and experimental results were in excellent agreement. However, to achieve this, ESA places greater demands on detector performance than DSA in terms of detector quantum efficiency (DQE), read noise, and scatter.

### Conclusion

With emerging detector technology and X‐ray system designs, it is now possible to obtain iodine‐specific images for ESA with similar image quality to that of DSA. ESA has the potential to be used for background removal in cardiac imaging and other applications, where DSA cannot be used.

SA‐B‐BRD‐02

## Commissioning of 2D/3D Matching on a Varian TrueBeam

J Dorand,^*^ D Ellerbusch, L Fong de los Santos, J Ma, D Pafundi

*Mayo Clinic, Rochester, MN, USA*

### Purpose

To commission the Varian TrueBeam 2D/3D six degree of freedom (6DoF) registration algorithm. 2D/3D matching uses an orthogonal 2D image pair matched to projections calculated from a 3D dataset.

### Methods

MIMI, Brainlab Pelvis, and RANDO C‐Spine phantoms were used for commissioning. 6DoF CBCT registration was used as the gold standard, with absolute maximum differences computed between 6DoF 2D/3D, and CBCT automatch results. Automatch algorithm configuration parameters were investigated and compared to Varian‐suggested parameters using the MIMI phantom. The Varian‐suggested parameters were used to test variations in reference CT slice thickness, acquisition angle, kV/kV vs. MV/kV, ROI selection, and the inclusion of an additional step in the matching algorithm process.

### Results

Varian‐suggested matching algorithm configuration parameters, along with additional parameter set definitions, yielded maximum absolute differences of 0.5 mm/0.6° and 0.8 mm/0.8°, respectively. Reference CT slice thicknesses of 1 mm and 2 mm for all three phantom geometries had maximum absolute differences of 0.4 mm/0.4° and 0.5 mm/0.3°, respectively. AP/Lat and posterior oblique acquisition angles yielded maximum absolute differences of 0.5 mm/0.4° and 0.8 mm/0.7°, respectively. Utilizing the MIMI and Brainlab pelvis phantoms, kV/kV vs. MV/kV results were 0.5 mm/0.3° compared to 0.7 mm/1.7°, respectively. RANDO C‐Spine ROIs of one vertebral body vs. three vertebral bodies yielded results of 0.7 mm/1.3° and 0.2 mm/0.7°, respectively, and the inclusion of an additional step in the matching algorithm resulted in differences of 0.1 mm/0.2° compared to 0.2 mm/0.7°.

### Conclusion

The 6DoF 2D/3D registration algorithm was determined to perform as well as the 6DoF CBCT algorithm for the rigid phantom geometries tested. ROI selection was found to have the greatest impact on differences between 2D/3D and CBCT matching results. A prospective clinical pilot of head and neck patients will test the efficacy of 6DoF 2D/3D registration on nonrigid anatomy.

SA‐B‐BRD‐03

## Comparability of Three Output Prediction Models for a Compact Passively Scattered Proton Therapy System

S Ferguson,^1*^ Y Chen,^1^ C Ferreira,^1^ M Islam,^1^ V Keeling,^2^ A Lau,^1^ S Ahmad,^1^ H Jin^1^

*Oklahoma Univ. Health Science Ctr.,^1^ Oklahoma City, OK, CARTI, Inc.,^2^ Little Rock, AR, USA*

### Purpose

To investigate comparability of three output (cGy/MU) prediction models for a compact passively scattered proton therapy system.

### Methods

Two published output prediction models were commissioned for our Mevion S250 proton therapy system: model (1) is a correction‐based model (Sahoo et al. Med Phys. 2008;35:5088‐97) and model (2) is a semi‐analytical model as a function of r=(R−M)/M (Kooy et al. PMB. 2005;50:5487‐56, 2005). The function r was modified to $\left((R^{\prime }-0.31)-0.81\times M^{\prime })/(0.81\times M^{\prime }\right)$ to convert the theoretical range R (distal 100% dose) and modulation M (distal 100% dose‐to‐proximal 100% dose) to the Mevion definition (R': distal 90% dose, M': distal 90% dose‐to‐proximal 95% dose). In addition, another quartic polynomial model (3) was newly developed based on the r. The outputs of 112 combinations of range R and modulation M covering the 24 options were measured. Each model's predicted output was compared to the measured output. In addition, outputs predicted by each model were also compared against each other using the Student's *t*‐test.

### Results

For the total dataset, the percent differences between predicted (P) using the three different models and measured (M) outputs ((P−M)/M×100%) were within ±3%. The average differences (± SD) were −0.02%±1.06%, 0.03%±1.16%, and −0.17%±1.27% for model (1), (2), and (3), respectively. The p‐values of the *t*‐test were 0.5869 (model (1) vs. (2)), 0.0725 (model (1) vs. (3)), and 0.0294 (model (2) vs. (3)). In general, the difference is greater as r becomes smaller for all the models.

### Conclusion

For all the options, all three models have clinically acceptable prediction. The difference between model (1) and model (2) (or model (3)) is statistically insignificant (p>0.07). Care should be taken when a small r of R and M combination is used. It is concluded that the models can comparably be used for the compact passively scattered proton therapy system.

SA‐B‐BRD‐04

## Effective and Efficient Grid Therapy Using High‐Dose‐Rate Flattening Filter‐Free Beam and Multileaf Collimator

M Liu,^*^ N Wen, F Siddiqui, I Chetty, B Zhao

*Henry Ford Health System, Detroit, MI, USA*

### Purpose

Treating bulky tumors with grid therapy (GT) has demonstrated high response rates in combination with conventionally fractionated external beam radiotherapy. MLC‐based GT (MLC‐GT) is becoming more popular because of its technical convenience and comparable dosimetric properties with the Cerrobend GT block. However, long delivery time (>15 min), with consequent increased risk of intrafraction motion, is a major disadvantage of conventional MLC‐GT. In this study, by taking the advantage of high dose rate of flattening filter‐free (FFF) beams and the beam automation feature of Varian TrueBeam system, we developed a GT technique with similar dosimetric characteristics, but more efficient delivery compared to conventional MLC‐GT.

### Methods

Two‐hundred 5 mm×5 mm grids were shaped by 40 central 5 mm leaves of a Millennium120 MLC. The distance between adjacent grids was 1 cm. A field‐in‐field 2D plan with 5 fields (3 cm×20 cm field size) was generated using 10XFFF beam (dose rate = 2400 MU/min) in the Eclipse treatment planning system (AAA‐v11) with prescription of 15 Gy to the grid at 1.5 cm depth. Each field contained two rows of grids. Doses were verified at depths of 1.5 cm, 5 cm, and 10 cm with calibrated Gafchromic EBT3 films in a 20 cm think, 30 cm×30 cm Solid Water phantom. The measured doses were compared to the Eclipse planar doses. Ten points were selected randomly to quantify the difference between the delivered and calculated doses.

### Results

The valley‐to‐peak dose ratio at the three depths was approximately 20%, which are very similar to published results. Film dosimetry revealed good agreement between the delivered and calculated dose. The overall gamma passing rates were >95% (5%, 1 mm). The point dose differences were 8.7%±2.5%, 5.8%±1.4%, and 4.9%±1.3% at depths of 1.5 cm, 5 cm, and 10 cm, respectively. The delivery time was 7 min.

### Conclusion

FFF beam combined with MLC and automation can provide effective and efficient GT for the treatment of bulky tumors.

The work was partially supported by a research scholar grant, RSG‐15‐137‐01‐CCE from the American Cancer Society.

SA‐B‐BRD‐05

## Evaluation of Electron Monte Carlo Dose Calculation of RayStation Treatment Planning System in Heterogeneous Media

S Mossahebi,^*^ M Lin, S Chen, H Xu, M Guerrero

*University of Maryland School of Medicine, Baltimore, MD, USA*

### Purpose

To evaluate the dosimetric performance of the Electron Monte Carlo (EMC) algorithm of RayStation treatment planning system (v.4.5).

### Methods

The dose calculation of RayStation TPS was compared with ion chamber, OSLD, and EBT2 film measurements for straight incidence and with EBT2 film measurements for oblique incidence in heterogeneous, multilayer phantoms to mimic the chest wall irradiation conditions. Both setups were irradiated (200 MUs) with 6, 9, and 16 MeV electron beams. For straight incidence, films were placed at the top polystyrene‐cork interface with two OSLDs around the center and the Farmer‐type ion chamber was positioned at the center of the bottom polystyrene layer. The phantom was irradiated with an open field and $3\times 6\;{\text{cm}}^{2}$ cut‐out at SSD 100 cm. For oblique incidence, films were placed vertically between two phantom sets. A $3\times 6\;{\text{cm}}^{2}$ cut‐out was irritated at gantry angles 0°, 10°, 20°, and 45°. Extended SSD (110 cm) was employed for 20° and 45°. The RayStation dose calculations were compared with the measurements for each energy and field size combination.

### Results

Agreements within 3% and 4% were found between TPS calculations vs. OSLD (high‐dose region) and ion chamber (low‐dose region) measurements, respectively. In the straight incidence, gamma analysis (3 mm/3%) showed an excellent agreement passing rate (>96%) between RayStation and film for all energies. In the small angle oblique incidence, >91% passing rate was observed at the high‐dose region for 6 and 9 MeV beams comparing to >82% for 16 MeV However, a poor agreement was observed for high angles and extended SSD for all energies (64%–81%).

### Conclusion

The Electron Monte Carlo algorithm of RayStation treatment planning system (v.4.5) is clinically acceptable for standard treatment and small angle incidence in heterogeneous treatment conditions. Caution should be paid when large angle incidence and extended SSD are utilized.

SA‐B‐BRD‐06

## Improvement of Tumor Localization in Cone‐Beam CT Using an Optical Tracking System

B Barraclough,^1*^ J Li,^2^ C Park,^2^ C Liu,^2^ S Lebron,^1^ G Yan^2^

*J. Crayton Pruitt Family Department of Biomedical Engineering,^1^ University of Florida, Gainesville, FL, Department of Radiation Oncology,^2^ University of Florida, Gainesville, FL, USA*

### Purpose

In cone‐beam CT (CBCT)‐guided external radiotherapy, baseline drifts in respiration‐induced tumor motions negatively affect CBCT image quality and tumor localization accuracy. The purpose of this work is to use an in‐house optical tracking system (OTS) to detect baseline drifts and develop correction strategies.

### Methods

The OTS consists of a pair of Polaris CCD cameras. It tracks an infrared reflective marker affixed on patients at 15 Hz throughout the treatment. State vectors in 2D augmented state space were formed with adjacent OTS signals. The vectors within a moving window were fitted to an elliptical shape prior, from which the centers of motion and baseline drifts were monitored. Individual CBCT projections acquired prior to baseline drifts were shifted accordingly in the 2D projection space to compensate for the drifts. The FDK algorithm was used to reconstruct the 3D CBCT volume data. A 4D motion phantom with a built‐in spherical tumor (3 cm diameter) was used to validate the algorithm. A regular sinusoidal pattern (2 cm peak‐to‐peak amplitude, 5 s period) was first simulated; then a 0.5 cm baseline drift was introduced at different stages of the CBCT scan (1/3, 2/3 way through and near end). The reconstructed tumor position was compared among the four breathing patterns.

### Results

Without correction, the reconstructed tumor was, respectively, 0.21, 0.33, and 0.42 cm away from the reference position. The later the drifts occurred in the scan, the larger the deviation in the tumor position. After correction, the deviation reduced to 0.08, 0.04, and 0.04 cm, respectively.

### Conclusion

Baseline drift in tumor motion during CBCT scanning negatively impacts tumor localization accuracy. Aided by an OTS, the proposed correction strategy can significantly improve the accuracy. Future work will examine its efficacy with different breathing patterns and drifts as well as its clinical impact in 4D CBCT.

SA‐B‐BRD‐07

## Improving Non‐MCO VMAT Planning in RayStation for Prostate Treatment with an Automated Knowledge‐Based Model Created Using a Database of Clinical MCO‐IMRT Plans

M. Young,^*^ L Vanbenthuysen, B Crawford, Y Wang

*Massachusetts General Hospital and Harvard Medical School, Boston, MA, USA*

### Purpose

To overcome the computational challenges of multicriteria optimization (MCO) for volumetric‐modulated arc therapy (VMAT) in RayStation using a knowledge‐based model constructed with sixty MCO‐based intensity‐modulated radiation therapy (IMRT) prostate plans.

### Methods

The same prescription was used for the IMRT and VMAT plans −50.4 Gy to PTV5040 ((expanded from prostate+proximal seminal vesicles)) followed by a boost of 28.8 Gy to PTV7920 (expanded from prostate). The dose‐volume histogram (DVH) for each target and organ at risk (OAR) was averaged over the MCO‐IMRT plans. The average DVH for each structure was used to calculate model constraints (upper range) and objectives (lower range) at select dose‐volume points. The model was implemented to guide non‐MCO VMAT optimization for 10 randomly‐selected clinical VMAT prostate plans. Each plan previously required an expert planner to initially generate a patient‐specific MCO‐IMRT plan to inform non‐MCO VMAT plan optimization. Model‐generated plan quality was compared to that of the planner‐generated plan using homogeneity index (HI) and conformity number (CI) for PTV5040, PTV7920, CTV5040, and CTV7920, and multiple DVH indices for bladder, rectum, anterior rectal half, posterior rectal half, femurs, and penile bulb. Statistical significance was assessed using the Wilcoxon ranked‐sum test.

### Results

All model‐generated plans achieved prescribed target coverage and satisfied OAR sparing per QUANTEC protocol. The model‐generated plans showed statistically significant improvement on target coverage, with HI decreased by 27.9%, 15.0%, and 21.2%, for PTV5040, PTV7920, and CTV7920, respectively. The model‐generated plans also showed statistically significant sparing improvements on the bladder, rectum, and femurs. The most improved OAR sparing was observed for Dmean and D2 of the bladder, which were reduced by 2.0±2.6 and 5.7±0.7 Gy, respectively.

### Conclusion

The automated, knowledge‐based model used an MCO‐IMRT plan database to substantially improve VMAT prostate planning plan quality over the manual VMAT planning technique in RayStation.

SA‐B‐BRD‐08

## Initial Experience with Pinnacle^3^ Autoplanning on Field Matching

S Wang,^1*^ D Zheng,^1^ W Zhen,^1^ Y Lei,^1^ X Zhu,^1^ X NI,^2^ C Enke,^1^ S Zhou^1^

*University of Nebraska Medical Center,^1^ Omaha, NE, USA, The First Hospital of Longyan Affiliated to Fujian Medical University,^2^ Longyan, Fujian, China*

### Purpose

Pinnacle^3^ autoplanning (AP) was introduced to improve labor‐intensive planning process and plan quality consistency. It utilizes progressive optimization to create planning structures, based on anatomical relationships among the PTVs and OARs, and iteratively tune the planning objectives during optimization. Our study evaluated AP planning on field matching between upper neck IMRT and lower neck 3D CRT fields, a more challenging problem for IMRT planning.

### Methods

Twelve patients treated with matching upper neck IMRT and lower neck anterior—posterior opposing fields were studied retrospectively. A generic AP technique was created by retrospectively sampling the institutional planning objectives of target volumes and anatomical OARs, excluding planning structures created by the planners. The AP composite plans with AP IMRT fields and clinical lower neck 3D CRT beams were compared to the clinical composite plans used for treatments. PTV coverage in the matching region, mean doses to the parotids, maximum doses to the spinal cord and brainstems, and plan conformity (prescription dose spillage) were evaluated. Mann‐Whitney U‐Test was used to compare between AP and clinical plans.

### Results

Overall PTV coverage were comparable, but AP plans achieved significantly better PTV coverage in the matching area, and significantly lower mean dose to parotids and max dose to brainstem (p<0.05). The max dose to spinal cord was on average lower in the AP plans, but not statistically significant. On the other hand, the prescription isodose volumes outside the PTVs were significantly larger in the AP plans, indicating inferior conformity in the AP plans than the clinical plans.

### Conclusion

With an expedited planning process with much less human‐thought process, AP results in better PTV coverage in the matching area and better OAR sparing for head‐and‐neck patients treated with combined IMRT and 3D CRT fields. Further improvement is necessary to achieve better plan conformity.

SA‐B‐BRD‐09

## Linear Accelerator Mechanical Quality Assurance Using a Calibrated Camera

D Robertson

*The University of Texas MD Anderson Cancer Center, Houston, TX, USA*

### Purpose

Monthly and annual mechanical quality assurance for linear accelerators (linacs) tests the spatial positioning of components that are essential for accurate delivery of radiation therapy. However, the tools used to perform these measurements are either low‐precision (e.g., graph paper or spirit level) or complex and labor‐intensive (e.g., Radac device). The purpose of this study is to evaluate the use of a simple calibrated camera system for precise, quantitative measurement of linear accelerator mechanical systems.

### Methods

A 1200×1600 pixel CCD camera was calibrated using multiple images of a planar checkerboard pattern and processed with an open‐source camera calibration toolbox. The camera was placed on the treatment couch of a Varian TrueBeam linac so that the camera field of view encompassed the light field projected on a white surface placed at 100 cm source‐surface distance. The spatial accuracy of the images was verified using images of a ruler. Images of the linac light field were used to measure mechanical parameters including: collimator rotation, optical distance indicator (ODI) accuracy, symmetric and asymmetric jaw positioning, multileaf collimator (MLC) positioning, couch motion, and localizing laser position.

### Results

The effective resolution of the calibrated camera was 0.3 mm at the center of the images of the linac light field. Interpolation of the light field penumbra, crosshair, laser, and ODI profiles enabled measurements of the jaw, MLC, laser, ODI, and couch lateral and longitudinal position with 0.1 mm precision. The angular position of the collimator was measured within 0.1°. The measurements agreed with historical records of mechanical quality assurance tests for the evaluated linac.

### Conclusion

A simple calibrated camera system can be used to precisely measure many of the mechanical properties of medical linacs. This approach provides precise, quantitative measurements that can be used to track the mechanical performance of linac components over time.

SA‐B‐BRD‐10

## Monte Carlo Study of 3D Dose Distributions for COMS Eye Plaques

J Gloss,^*^ C Watchman

*University of Arizona, Tucson, AZ, USA*

### Purpose

The purpose of our study is to investigate heterogeneity correction factors for three different vendor seed models using a Monte Carlo transport code to compute the three‐dimensional dose distributions for COMS eye plaques as recommended by TG‐186.

### Methods

Monte Carlo N‐Particle transport code (MCNP5) was used for Monte Carlo simulations. Three different vendor models were investigated: Amersham Oncoseed 6711, Best Medical 2301, and IsoAid IAI‐125. Seed characteristics were benchmarked against TG‐43 and existing literature to verify the seed models. Simulations of COMS plaques ranging from 10 mm to 22 mm were evaluated for three‐dimensional dose distributions in a heterogeneous medium and a homogeneous water medium. Heterogeneity correction factors were calculated, and off‐axis factors were applied to a sample of 10 patient plans in order to evaluate the impact of the calculated heterogeneity correction.

### Results

Heterogeneity correction factors were computed for COMS plaque sizes ranging from 10 mm‐22 mm. Central axis dose correction varied from 10%–12% up to 1 cm which matches previously published data. Off‐axis peripheral tumor doses varied up to 15% lower for the heterogeneous computation compared to the homogeneous computation.

### Conclusion

Monte Carlo computed dose distributions in heterogeneous media provide a more accurate dose calculation than current methods. Heterogeneity correction factors should be used to evaluate the off‐axis doses for COMS eye plaques. While central axis doses vary 10%–12%, peripheral doses evaluated from the patient sample indicate reduction in off‐axis dose targets. Consequently the use of heterogeneity correction factors results in improved accuracy in the calculation of dose to structures at risk.

SA‐B‐BRD‐11

## Unified Database for Rejected Image Analysis Across Multiple Vendors in Radiography

K Little,^*^ L Liu, K Haas, A Sanchez, I Reiser, Z Lu

*The University of Chicago, Chicago, IL, USA*

### Purpose

Rejected image analysis is an important part of a QA program to minimize patient radiation dose and maintain quality in radiography. A clinical reject is an X‐ray acquisition of a patient that is discarded without being reviewed by a radiologist. However, variations in acquisition reporting by different equipment vendors make reject analysis difficult. Our adult imaging areas use six DR and CR models from three vendors. A centralized reject database and reporting dashboard were developed to allow for consistent clinical reject analysis across all radiographic equipment at our facility.

### Methods

Acquisition reports were retrieved monthly for each unit and imported into an SQL database. The database was interfaced with RIS to associate each record with the performing technologist. We developed counting rules for each model to ensure that multiple records for a single acquisition (as in the case of dual‐energy acquisitions) were not duplicated. The various vendor‐provided clinical reject reasons were mapped into one of five categories: incorrect technique, positioning/collimation, artifact/obstruction, patient motion, and other. Each vendor's varying anatomy descriptors were mapped to a unified terminology.

### Results

Data were analyzed based on reject reason, anatomy/view, clinical area, equipment, and staff. Interventions were developed to target the most frequent reject reason and the procedure with the highest rejects. Reject rates for each technologist were reported to the area manager to identify the need for individualized training. Initial clinical reject rates were found to be higher than expected based on available literature and generally higher for DR than for CR.

**Conclusion**

The monitoring of rejected images in radiography can be a complicated task when multiple manufacturers and models are used in the same department. A centralized database, repeat reason categorization, and anatomy/view mapping allow clinical reject data to be consistently analyzed and used for process improvement.

This work was supported in part by the RSNA/AAPM Imaging Physics Residency Program Grant.

**Therapy Symposium ‐ SAM Grand Ballroom D *QA Fundamentals: Applications of Multi‐dimensional Arrays***

SA‐C‐BRD‐00

## QA Fundamentals: Applications of Multi‐Dimensional Arrays

Moderator: Kyle Antes

*Presbyterian Healthcare System, Dallas, TX*

SA‐C‐BRD‐01

V. Feygelman, H. Lee

*Moffitt Cancer Center*

**Clinical Needs Driving Multi‐Dimensional Systems**

SA‐C‐BRD‐02

S. Stathakis

*CTRC at UTHSCSA*

**Leveraging Multi‐Dimensional Systems for IMRT QA**

SA‐C‐BRD‐03

T. Ritter

*The University of Michigan*

**Leveraging Multi‐Dimensional Systems for TG142**

Multidimensional dosimetry arrays can be useful tools for commissioning of the planning/delivery chain, linear accelerator quality assurance (QA), and patient‐specific end‐to‐end testing for intensity‐modulated radiation therapy (IMRT) and volumetric‐modulated arc therapy (VMAT) treatments. We will give an introduction to the available arrays on the market, examine differences between the ion chamber‐ and diode‐based arrays, review their fundamental properties, and provide recommendations on commissioning. We will describe the place of the multidimensional arrays in the overall QA system, including which dose analysis techniques should be used for applications such as assessing linear accelerator performance. The inherent limitations of the 2D arrays will be discussed in comparison with the quasi‐3D arrays. Applications to linear accelerator QA will include specific examples of multileaf collimator (MLC), output, profile, and energy checks, as well as performing postrepair testing and validation with detector arrays.

### Learning Objectives:

1. To learn the properties of multidimensional arrays, their strengths and their drawbacks.
2. To understand the proper use of multidimensional arrays in patient specific plan QA, including 2D vs. 3D array analysis.
3. To learn the difference between 3D dose reconstruction and direct application of quasi‐3D arrays.
4. To learn the use of multidimensional arrays in linear accelerator and treatment planning quality assurance including their use for TG‐142 QA.

**Diagnostic Symposium ‐ SAM Grand Ballroom A *Clinical MR Safety***

SA‐C‐BRA‐00

**Clinical MR Safety**

Moderator: Jeffrey Moirano

*University of Washington, Seattle, WA*

SA‐C‐BRA‐01

**Background ‐ Basis for MRI Safety Programs**

M. Amurao

*Columbia University*

SA‐C‐BRA‐02

**Model MRI Safety Program ‐ The Case Western Reserve University Experience**

D. Jordan

*University Hospitals Case Medical Center*

SA‐C‐BRA‐03

**Model MRI Safety Program ‐ The Mayo Clinic Experience**

H. Edmonson

*Mayo Clinic College of Medicine*

SA‐C‐BRA‐04

**Relevant Certifications for Medical Physicists**

M. Amurao

*Columbia University*

There has been a rapid increase in the number of reported MR‐related incidents in the past 15 years, which has precipitated the response by accrediting organizations to develop expanded MRI‐safety requirements.

The Medical Physicist plays a central role in the development, implementation, investigation, and regular review of the physical and organizational infrastructure that a site needs to maintain an MR‐safe environment.

This presentation discusses the approaches taken by several organizations — from a medical physicist's perspective — in the implementation of MR‐safe procedures, policies, and practices. The presentation will also touch on the salient points of MR‐safety topics relevant to accreditation, as well as the credentialing of MR‐safety personnel with an emphasis on the MR Safety Expert (MRSE) certification.

### Learning Objectives:

1. Understand the increased need for MR safety
2. Understand the key components in an MR safety program
3. Methods to implement and track MR safety programs in the clinic

Therapy Symposium ‐ SAM Grand Ballroom D *Why Gamma Isn't Enough: Revisiting Our Criteria and Interpretation of IMRT QA Results*

SA‐D‐BRD‐00

## Why Gamma Isn't Enough: Revisiting Our Criteria and Interpretation of IMRT QA Results

Moderator: Kyle Antes

*Presbyterian Healthcare System, Dallas, TX*

SA‐D‐BRD‐01

**Limitations of Gamma Analysis**

D. Low

*UCLA*

SA‐D‐BRD‐02

**Digging Into Our Toolbox: A Non‐Gamma Analysis Tools for IMRT**

J. O'Daniel

*Duke University Medical Center*

Gamma analysis has been an extensively used tool for dose distribution comparisons, most often employed for pretreatment patient specific quality assurance analysis. For convenience, dose distribution pass/fail comparisons have relied on the gamma failure rate, which has been shown to be insensitive to many clinically relevant dose delivery uncertainties. In this session, the gamma analysis tool will be discussed to show what it can and cannot do. A road map and hints will be discussed to go from where we are now to a practical tool or tools that can take the place of gamma failure rate analysis. Other patient‐specific QA analysis tools will be discussed, including three‐dimensional DVH analysis and multiplanar analysis.

### Learning Objectives:

1. To learn and be able to define what gamma analysis is and what it is not.
2. To understand the situations when gamma analyses may not be sensitive enough to catch clinically relevant dose delivery errors.
3. To understand when gamma analysis might be insufficiently sensitive and when this is acceptable and not acceptable.
4. To learn about other analysis tools that can be used that might be appropriate replacements for gamma analysis.

Diagnostic Symposium ‐ SAM Grand Ballroom A *X‐Ray Tube Design*

SA‐D‐BRA‐00

**X‐Ray Tube Design**

Moderator: Jeffrey Moirano

*University of Washington, Seattle, WA*

SA‐D‐BRA‐01

**History and Future of the X‐Ray Tube: Can We Do It Better?**

R. Behling

*Philips Medical Systems DMC GmbH*

In the vast majority of radiological sites for imaging and therapy, vacuum electronics devices are still generating bremsstrahlung, more than 120 years after Conrad Roentgen's discovery. Wouldn't it be time for a change? Where is the innovation? How do current X‐ray tubes function? How do they look like after dissection? What are the technical trends? Will we see X‐ray LEDs, compact X‐ray lasers or flat‐panel sources soon in medical imaging?

This lecture will try to raise understanding and provide answers. It will briefly touch on the physics of X‐ray generation, treat key characteristics of X‐ray sources, materials and components, manufacturing technology, cost drivers, and service aspects. Important quality parameters for comparison and selection of the most suitable source will be identified. Advice will be given to extend the service life. It will become evident that the term X‐ray tube understates the importance of well‐matched supporting generator electronics, which controls the very complex functions of modern diagnostic X‐ray sources.

### Learning Objectives:

1. Know tube design concepts and subcomponents
2. Understand the importance of well‐matched control electronics
3. While departing from outdated metrics, learn to compare tubes of current technology
4. Understand why X‐ray tubes fail early and how this can be avoided

Mr. Behling is an employee of Royal Philips

**SUNDAY, MARCH 6**

**Therapy Symposium ‐ SAM Grand Ballroom D *Translating AAPM Task Group 100 Into Our Clinics***

SU‐A‐BRD‐00

**Translating AAPM Task Group 100 Into Our Clinics**

Moderator: Jean Moran

*University Michigan Medical Center, Ann Arbor, MI*

SU‐A‐BRD‐01

**Applying Risk Analysis Techniques to Routine QA**

K. Smith

*Mary Bird Perkins Cancer Center*

SU‐A‐BRD‐02

**Applying Risk Analysis to High Risk Procedures**

T. Pawlicki

*UCSD Medical Center*

AAPM Task Group 100 recommends risk‐based analysis techniques, such as failure modes and effects analysis (FMEA), to improve quality control programs. Performance of an FMEA is a team effort, and the presentation will address performing FMEA from a group or collaborative approach and review the meaningful dialogue that is helpful when organizing such a task. Individuals involved should have an in‐depth knowledge of the process being evaluated and a working knowledge of the steps involved in the FMEA risk analysis. The team estimates failure modes, along with the probability of the occurrence of the failure, the likelihood of detecting the failure, and the impact on the patient. This session will report on the growing experience with applying FMEA to routine clinical physics duties in radiation therapy such as external‐beam treatment planning and linear accelerator quality assurance. It is useful to understand potential weaknesses and safety concerns for any clinical process that impacts the patient and therefore, risk analysis may be performed for any process, procedure or routine quality assurance check used in the clinic. FMEA can also be extremely beneficial when applied to high risk procedures such as stereotactic radiosurgery (SRS), stereotactic body radiation therapy (SBRT), and high‐dose‐rate brachytherapy. Examples will be shared for the two types of applications. In addition, environmental considerations, such as how to educate and create a group to tackle an FMEA, will be presented for single institution and multi‐institutional approaches.

### Learning Objectives:

1. Learn about how FMEA has been used to improve routine radiation therapy quality checks
2. Learn about how to apply FMEA tools to high‐risk clinical procedures
3. Learn about how to work with others to perform an FMEA within a department or with colleagues at other institutions

Sasa Mutic receives research funding from Varian. Peter Dunscombe is Director, TreatSafely, LLC; Director, Center for the Assessment of Radiological Sciences; and Occasional Consultant to IAEA and Varian.

**Mammography Symposium ‐ SAM Grand Ballroom A *Digital Breast Tomosynthesis***

SU‐A‐BRA‐00

**Digital Breast Tomosynthesis**

Moderator: Jessica Clements

*Kaiser Permanente, Los Angeles, CA*

SU‐A‐BRA‐01

K. Hulme

*The Cleveland Clinic*

**Unique Features of the Siemens Inspiration Digital Breast Tomosynthesis System**

SU‐A‐BRA‐02

T. Fisher

*Therapy Physics, Inc.*

**Unique Features of the GE SenoClaire Tomosynthesis System**

Two new digital breast tomosynthesis units have been approved by the FDA for clinical use in the United States in 2014 and 2015. The objective of this course is to provide the medical physicist an overview of the unique features of the GE Senoclaire and Siemens Inspriation Digital Breast Tomosynthesis systems, and how to perform the required medical physics tests for acceptance testing and review the process for submission to the FDA DBT certificate extension program. This program is designed for medical physicists who are familiar with testing GE and Siemens digital mammography systems and will build upon this knowledge.

### Learning Objectives:

1. Review of Senoclaire and Inspriation hardware and software features
2. Review the Medical Physicist's required tests
3. Review the requirements for FDA Certificate Extension Program submission
4. Review the Technologist QC program

**Diagnostic Symposium Grand Ballroom A Diagnostic Updates and Information Session with The Joint Commission**

SU‐B‐BRA‐00

## Diagnostic Updates and Information Session with The Joint Commission

Moderator: Dustin Gress

*MD Anderson Cancer Center, Houston, TX*

**SU‐B‐BRA‐01**

A. Browne

*The Joint Commission*

**Experience and Path Forward After Year 1 of New Requirements**

The Joint Commission (TJC) had their new Diagnostic Imaging Requirements go into effect July 1, 2015. This presentation, given by TJC, will provide background on how the new requirements evolved, discuss how the medical physicist provides value in the context of TJC's expectations, and allow generous time for questions and answers. TJC will also discuss their current and future initiatives that will impact medical imaging and physicists.

### Learning Objectives:

1. Understand genesis of new TJC Diagnostic Imaging Requirements;
2. Learn TJC expectations of medical physicist participation and performance under new Diagnostic Imaging Requirements; and
3. Become aware of ongoing and future TJC initiatives impacting the medical imaging and physicists.

**Professional Symposium ‐ SAM Grand Ballroom D *Total Quality in Radiation Therapy***

SU‐B‐BRD‐00

**Total Quality in Radiation Therapy**

Moderator: Brent Parker

*University Texas Medical Branch of Galveston, Galveston, TX*

SU‐B‐BRD‐01

**Strategies for Total Quality in Radiation Therapy ‐ Part I**

S. Hancock

*Southeast Missouri Hospital*

SU‐B‐BRD‐02

**Strategies for Total Quality in Radiation Therapy ‐ Part II**

U. Hancock

*Dale and Hancock Center*

The evolving role of the medical physicist in radiation therapy goes beyond application of physics and technology. The core responsibility of the physicist in radiation therapy is the assurance of quality. Beyond quality control testing of equipment, assurance of quality requires that people follow effective processes. But studies suggest that many physicists have a low aptitude for the social skills needed to effectively guide and influence neurotypical nonphysics staff members. A physicist's development of the needed skills often requires cognitive application of behavioral models that may be simply intuitive to neurotypicals. This presentation takes a broad view of the meaning of quality in radiation therapy, with a focus on the physicist's role in the pursuit of quality. The emphasis is on applicable models from fields that are typically not covered in a medical physicist's education and training. The strategic approaches to the pursuit of quality in radiation therapy draw from the fields of psychology, philosophy, industrial engineering, management, mathematical biology, and neuroscience.

This presentation covers the following strategies:

1. Tools, not rules — The added cost of the tools of today is justified by increased efficiency.
2. Delegate — Delegation of recurring tasks increases the value of both the physicist and the delegate employee. The Situational Leadership model is presented as a guide for effective delegation.
3. Process design and improvement — QC of equipment performance is not enough if people are not following effective processes.
4. Create a culture of Total Quality Management — Provide leadership and get started with Root Cause Analysis.
5. Incident Learning System — Identify and prioritize opportunities for improvement.
6. Experience Design — The patient's perceptual experience should be included in measures of quality.
7. The Opportunity, Influence, Impact cycle — You need opportunities for awareness and influence.
8. Customer‐supplier feedback — Equipment suppliers should be included in your quality improvement system.
9. Organizational strategies — A matrix management model can be an effective and efficient organizational scheme for medical physicists and dosimetrists.
10. Systems Theory model of organizational psychology — Influence staff without direct authority.
11. Medical physicist archetypes from 20th century American mythology — Be the town marshal, not the Lone Ranger.

### Learning Objectives:

1. Define the broad meaning of quality in the context of Total Quality Management, and its application in radiation therapy
2. Describe the physicist's role in the assurance of quality in radiation therapy; Demonstrate an understanding of 11 strategies for achieving quality.

Therapy Symposium Grand Ballroom D *Putting It All Together — Practical Advice on Implementing TG100*

SU‐C‐BRD‐00

**Putting It All Together — Practical Advice on Implementing TG100**

Moderator: Jean Moran

*Univ Michigan Medical Center, Ann Arbor, MI*

SU‐C‐BRD‐01

## Putting It All Together —Practical Advice on Implementing TG100

T. Pawlicki

*UCSD Medical Center*

SU‐C‐BRD‐02

**Putting It All Together — Practical Advice on Implementing TG100**

P. Dunscombe

*The University of Calgary*

SU‐C‐BRD‐03

**Putting It All Together — Practical Advice on Implementing TG100**

D. Brown

*University of California, San Diego*

This session provides hands on training on how to use tools of process mapping, failure modes and effects analysis (FMEA), and causal analysis for use as part of a comprehensive quality management program as recommended by AAPM Task Group 100. Key concepts will be introduced that help facilitate the use of these tools, as well as provide participants with what should be expected when using these tools. Teams will create a process map from patient entry into the treatment room through beam‐on, which will be followed by an FMEA of some steps in the process of patient setup for treatment. An example error will be shared and a causal analysis of the event will then be performed by each group. Finally, groups will determine mitigation strategies and revisit how those strategies impact the results of the FMEA to assess the effectiveness of those strategies. Throughout the session, teams will interact with a facilitator and then experiences will be shared with the full group.

### Learning Objectives:

1. Understand how to create a process map and use it as part of an FMEA
2. Learn how to apply causal analysis techniques to the investigation of an event
3. Understand how to use the risk priority numbers of an FMEA when assessing the potential effectiveness of mitigation strategies

**Professional Symposium ‐ SAM Grand Ballroom A *Dollars and Sense: Are We Overshielding Imaging Facilities***

SU‐C‐BRA‐00

## Dollars and Sense: Are We Overshielding Imaging Facilities

Moderator: Matthew Meineke

*Ohio State Univ, Columbus, OH*

SU‐C‐BRA‐01

R. Marsh

*University of Colorado School of Medicine*

**An Analysis of Shielding Design in Medical Imaging: Are We Overshielding? Part I**

SU‐C‐BRA‐02

B. Murray

*Ohio Medical Physics Consulting, LLC*

**An Analysis of Shielding Design in Medical Imaging: Are We Overshielding? Part II**

Diagnostic imaging and nuclear medicine play a vital role in the diagnosis and treatment of disease. While an individual patient clearly receives a direct benefit from direct exposure to medical radiation, clinical medical physicists are often tasked with shielding employees and the public from this same radiation.

Determination of shielding requirements for diagnostic imaging and nuclear medicine facilities is predominantly guided by NCRP Report 147 and AAPM Report 108. These documents have been instrumental in standardizing the way in which shielding design is performed. In the decade since their publication, however, the basis for the reports' recommendations have been forgotten, and shielding is often approached as a methodical process rather than a careful analysis of risks and benefits.

Shielding design is a balance of competing factors — radiation exposure to staff and the public, clinical workflow, and building costs. This session will provide a critical evaluation of current shielding practices, including a review of the basis for the recommendations made by NCRP and AAPM, and a cost‐benefit analysis of current methodology. The speakers will address shielding for diagnostic imaging and nuclear medicine facilities.

### Learning Objectives:

1. Understand the assumptions made by NCRP Report 147 and AAPM Report 108 in establishing shielding guidance
2. Understand the monetary cost associated with shielding diagnostic imaging and nuclear medicine facilities
3. Understand the benefits provided to employees and the public from shielding diagnostic imaging and nuclear medicine facilities

Bryon Murray, MS, DABR is a paid consultant for shielding calculations by NELCO.

**Therapy Symposium ‐ SAM Grand Ballroom D *The National Landscape of Radiation Therapy Safety Efforts***

SU‐D‐BRD‐00

## The National Landscape of Radiation Therapy Safety Efforts

Moderator: Jean Moran

*University Michigan Medical Center, Ann Arbor, MI*

SU‐D‐BRD‐01

J. Johnson

*UT MD Anderson Cancer Center*

**Improving Patient Safety: Contributions From the Work Group on Prevention of Errors in Radiation Oncology**

SU‐D‐BRD‐02

G. Ezzell

*Mayo Clinic Arizona*

**Report Card on RO‐ILS: What Have We Learned?**

The national landscape for safety in radiation therapy is continually improving and AAPM has had efforts central to those improvements. AAPM and ASTRO launched the Radiation Oncology‐Incident Learning System in July, 2014. As of November 2015, 80 institutions representing 163 facilities have joined with more than 1060 events reported. This session will share the current state of national incident learning with the RO‐ILS system, the event review process, how various institutions use the system differently, and how useful information is being returned to the community. Lessons learned from the first 18 months of use will be discussed and examples given about failure modes and best practices reported and how to improve data submission to facilitate such learning.

The Work Group on Prevention of Errors (WGPE) has led several of the national efforts to improve patient safety by providing resources that are readily available to AAPM members for use. Resources include the consensus recommendations for incident learning systems, the Safety Profile Assessment (SPA) tool, Task Group 230 — MPPG 4.a on safety checklists, and numerous educational sessions available in the AAPM virtual library. New initiatives focus on supporting the Task Group 100 rollout, developing Task Group 275 on strategies for effective physics plan and chart review, examining safety barriers and their effectiveness from RO‐ILS data, and developing policy and procedure templates built from SPA results. AAPM will continue to focus on safety improvements in patient care.

### Learning Objectives:

1. Learn how to create a more complete event narrative that facilitates independent review
2. Learn about incidents reported nationally that can lead to improvements within your own clinic
3. Learn about safety efforts by the WGPE and how you can use these tools within your own clinic

**Mammography Symposium ‐ SAM Grand Ballroom A *Stereotactic Breast Biopsy and Molecular Breast Imaging***

SU‐D‐BRA‐00

## Stereotactic Breast Biopsy and Molecular Breast Imaging

Moderator: Jessica Clements

*Kaiser Permanente, Los Angeles, CA*

SU‐D‐BRA‐01

S. Kappadath

*UT MD Anderson Cancer Center*

**Molecular Breast Imaging: History and Recent Developments**

Molecular Breast Imaging (MBI) is an emerging breast‐specific nuclear medicine imaging technique that uses a small field‐of‐view, dual‐headed, semiconductor‐based gamma camera in a mammographic configuration specifically designed to obtain high resolution images of 99mTc‐sestamibi uptake in the breast. Modern MBI systems incorporate a number of technological innovations that leads to improved image quality and lower radiation doses. MBI is poised to make substantial contributions in the detection and managements of patients with breast cancer.

### Learning Objectives:

1. To become familiar with the historical developments on MBI
2. To understand the technological advances in MBI systems that leads to improved image quality and lower radiation doses
3. To become familiar with clinical role of MBI for patients with breast cancer

**MONDAY, MARCH 7**

**Therapy Symposium ‐ SAM Grand Ballroom D**

***Challenges in Electronic Recordkeeping***

MO‐A‐BRD‐00

## Challenges in Electronic Recordkeeping

Moderator: Brian Wang

*University Louisville, Louisville, KY*

MO‐A‐BRD‐01

J. Mechalakos

*Memorial Sloan‐Kettering Cancer Center*

**Overview of TG262 on Electronic Record Keeping & Clinical Experience with Aria**

MO‐A‐BRD‐02

B. Salter

*University Utah*

**Clinical Experience with MOSAIQ for Electronic Record Keeping**

MO‐A‐BRD‐03

S. Dieterich

*UC Davis Medical Center*

**Electronic Charting for Brachytherapy and NRC Regulations**

Over the last several years there has been an effort to utilize electronic recordkeeping in radiation oncology, mostly motivated by government regulations and budgetary considerations. A number of commercially available electronic medical record systems have been developed for radiation oncology. However, the implementation of these systems for the different aspects of information for radiation oncology (e.g., imaging, treatment planning, and delivery, on treatment and follow‐up data) is not always straightforward, often involving the development of internal processes to make the integration of these systems into the clinic more seamless and robust. Thus, the transition from a “paperful” to an electronic environment can be challenging, with the ultimate benefit of maintaining the integrity and accessibility of patient information. In this session we will provide an overview of TG‐262, highlight clinical experiences with common commercially available systems for electronic record keeping, as well as provide insight on how to perform electronic charting for brachytherapy.

### Learning Objectives:

1. To review the charges and progress of TG262
2. To discuss the practical uses and challenges of using Aria
3. To discuss the practical uses and challenges of using MOSAIQ
4. To present the implementation of electronic charting for brachytherapy and to review related NRC regulations

**Mammography Symposium — SAM Grand Ballroom A**

**New Concepts in Breast Imaging**

MO‐A‐BRA‐00

## New Concepts in Breast Imaging

Moderator: Jessica Clements

*Kaiser Permanente, Los Angeles, CA*

MO‐A‐BRA‐01

J. Boone

*UC Davis Medical Center*

**Changing Perceptions and Updated Methods for Mammography Dosimetry — Part I**

MO‐A‐BRA‐02

A. Hernandez

*University of California*

**Changing Perceptions and Updated Methods for Mammography Dosimetry — Part II**

MO‐A‐BRA‐03

J. Cord

*Kaiser Permanente*

**Dense Breasts, Risk Stratification, DCIS Controversy & Genetic Based Risk Stratification — The Road to Customized Care**

## CHANGING PERCEPTIONS AND UPDATED METHODS FOR MAMMOGRAPHY DOSIMETRY

Mammography as a technology is changing, and previous dogma about breast geometry have changed, as well. Consequently, there is a need for change in mammography dosimetry methods and tools. X‐ray tube anodes in mammography have traditionally been molybdenum or rhodium, but the use of tungsten anodes has emerged on newer mammography systems, with different filter materials such as aluminum, silver, palladium, etc. Dose coefficients (DgN values) need to be updated to accommodate these new spectra. Three‐dimensional breast imaging modalities, such as MRI and breast CT, have generated new a understanding about the geometry of the breast, including a thinner skin layer (1.5 mm versus 4.0 mm), a dramatic change in the average breast density (∼15% versus 50%), and a better description of the 3D distribution of glandular tissue in the breast (heterogeneous versus homogeneous) with a concomitant 30% reduction in DgN values which consider real heterogeneous breast geometry. This symposium will describe new X‐ray system designs which impact dose assessment, and will also demonstrate how perceptions of breast geometry have changed using breast CT datasets. Using these new data, methods and tools for more accurate assessment of mean glandular dose will be described. A complete set of updated references for breast dosimetry will be provided to attendees.

### Learning Objectives:

1. Previous assumptions about breast geometry have changed with the advent of 3D breast imaging using both MRI and breast CT.
2. New data have led to a new understanding in the range of volume breast density, and in the normalized glandular dose coefficients (DgN) values used in computing breast dose.
3. Some new mammography systems use different X‐ray target materials than the traditional Mo and Rh anodes, and practitioners need to make use of newly available tables for these more exotic anode/filter combinations.
4. The attendee should be able to identify new trends in breast geometry and mammography system design, and will be provided new DgN tables (as references) for producing more accurate MGD estimates in clinical practice.

## DENSE BREASTS, RISK STRATIFICATION, DCIS CONTROVERSY & GENETIC BASED RISK STRATIFICATION — THE ROAD TO CUSTOMIZED CARE

Mammography has been the standard for breast cancer mass population screening of asymptomatic women for decades. Our supplemental technologies are evolving and our understanding of genetic risk is constantly improving. Many modalities are competing with the status quo, and the benefits of the current screening regimen are constantly being reevaluated and challenged. To complicate matters, our legislative bodies have responded with political elements and confusing messaging. All the while we seem to be diagnosing more breast cancer than ever! This session will take a journey through the various controversies showing the ever changing landscape of breast cancer screening highlighting the current controversies and their origins. We will then attempt to evaluate future directions and personalized screening potentials.

### Learning Objectives:

1. Understand verbiage of “dense breast legislation”
2. Understand some of the controversy associated with aggressive imaging and overdiagnosis
3. Be able to discuss weaknesses and strengths of alternative supplemental imaging modalities
4. Better understand genetic predisposition and risk‐specific screening options
5. Learn some of the current imaging recommendations for high‐risk women
6. Gain insight on evolving technologies

**Therapy Symposium ‐ SAM Grand Ballroom D**

**Permanent Interstitial Brachytherapy for Prostate Cancer**

MO‐B‐BRD‐00

## Permanent Interstitial Brachytherapy for Prostate Cancer

Moderator: Brian Wang

*University Louisville, Louisville, KY*

MO‐B‐BRD‐01

J. Tward

*University of Utah*

**A Physician's Perspective and Experience**

MO‐B‐BRD‐02

V. Narayana

*Providence Cancer Center*

**Using Task Group 137 to Prescribe and Report Dose**

Permanent interstitial brachytherapy has become the standard of care for selected prostate cancer patients in recent years. The techniques for implantation have evolved in several different forms and new radioactive sources have been explored in clinical practice. Lessons can be learned from reported incidents including the importance of implementing a formal and safe program. This session will include discussions from both physician and physicist perspectives. The physician will describe the clinical need of the procedure and share his rich experience on this procedure. The physicist will review the AAPM guidelines of TG137 for dose prescription for routine patient treatment, clinical trials, and for treatment planning software developers. We will describe the current recommendations on using D90 and V100 as the primary quantities and discuss more specific guidelines on the use of the imaging modalities and the timing of the imaging.

### Learning Objectives:

1. To understand the clinical need of permanent interstitial brachytherapy for prostate cancer
2. To learn the implantation and image guidance techniques
3. To understand the physics considerations and advantages and disadvantages of different radioactive sources
4. To understand the TG137 recommendation for dose prescription

**Diagnostic Symposium Grand Ballroom A**

**Nuclear Medicine & PET**

MO‐B‐BRA‐00

**Nuclear Medicine & PET**

Moderator: Dustin Gress

*MD Anderson Cancer Center, Houston, TX*

MO‐B‐BRA‐01

S. Schwarz

*Washington University*

**PET Radiopharmaceuticals: Past, Present & Future**

This session will review the basic physics and operation of cyclotrons in the context of their role in radiopharmaceutical production. The history of PET radiopharmaceuticals will be discussed, along with the FDA approval process for them. The session will wrap up with detailed discussion of recent advances and future possibilities in the field of PET radiopharmaceuticals.

### Learning Objectives:

1. Review basic physics, operation, and role of cyclotrons with respect to radiopharmaceuticals;
2. Understand history of PET radiopharmaceuticals and how they become approved by the FDA for clinical use; and
3. Learn where leaders in the field see opportunities for enhanced patient care via innovation in PET radiopharmaceuticals.

**Diagnostic Symposium ‐ SAM Grand Ballroom A**

**Nuclear Medicine & PET**

MO‐B‐BRA‐00

**Nuclear Medicine & PET**

Moderator: Dustin Gress

*MD Anderson Cancer Center, Houston, TX*

MO‐B‐BRA‐01

S. Kappadath

*UT MD Anderson Cancer Center*

**Gamma Camera and SPECT Basics & Performance**

Single‐photon emission computed tomography (SPECT), together with computed tomography (CT), commonly referred to as SPECT/CT, are rapidly becoming a mainstream Nuclear Medicine imaging modality. SPECT/CT scanners sequentially acquire both anatomic and functional information that is complementary in a single examination. Another important feature of SPECT/CT imaging is the ability to model the physics of the detector system and correct the nuclear emission images for attenuation and photon scatter to obtain more accurate image data. This lecture will review the physics principles underlying SPECT and SPECT/CT imaging, discuss quality assurance of SPECT/CT systems, and present several examples of the clinical application of SPECT/CT.

### Learning Objectives:

1. Review the physics principles underlying SPECT/CT image acquisition and reconstruction;
2. Understand the quality assurance procedures specific to SPECT/CT systems; and
3. Become familiar with clinical applications of SPECT/CT imaging.

**Professional Symposium Grand Ballroom D**

**Regulatory Update**

MO‐C‐BRD‐00

**Regulatory Update**

Moderator: Lynne Fairobent

*AAPM, Alexandria, VA*

MO‐C‐BRD‐01

R. Martin

*AAPM*

**Behind the Doors: A Perspective on Medical Event Reporting**

MO‐C‐BRD‐02

B. Finerfrock

*Capital Associates, Inc.*

**Washington Health Policy Update — What's Next?**

MO‐C‐BRD‐03

L. Fairobent

*AAPM*

**Challenges in Complying with Regulations**

Regulation and legislation impact every medical physicist's day‐to‐day performance. However, the process and requirements are not always clear. This session will first will review what is behind the doors of current medical event reporting requirements and trends. We will look at how the Institute of Medicine report “To Err Is Human: Building a Safer Health System” (2000) raised public awareness of medical events and safety. We will address the *Patient Safety and Quality Improvement Act* (2005), which spurred growth of incident learning systems, including the Radiation Oncology Incident Learning System (RO‐ILS), and talk about current state regulations, some of which employ graded penalties based on levels of noncompliance.

Secondly, this session will also address recent changes adopted by Congress that will affect how health‐care services are covered and paid for in the future. The presentation will discuss and contrast the differences between the current fee‐for‐service system and alternative payment models, such as episodes of care and bundled payments, and what these may mean for patients and providers. In addition, the presentation will also discuss the MIPS initiative and how this is different from Alternative Payment Models, the transition timetable for moving to MIPS/APMs, and what providers need to do to prepare for these changes. Finally, this session will address recent changes in the regulatory environment, such as the impact of accreditation and how it relates to federal and state mandates, to include a look ahead to the potential impact the 2016 Presidential elections might have.

**Therapy Symposium ‐ SAM Grand Ballroom D**

***Task Group 210: Linac Fundamentals of Conventional Linac Acceptance Testing***

MO‐D‐BRD‐00

## Task Group 210: Linac Fundamentals of Conventional Linac Acceptance Testing

Moderator: Brian Wang

*Univ Louisville, Louisville, KY*

MO‐D‐BRD‐01

D. Rangaraj

*Baylor Scott & White Health*

**Task Group 210: Linac Fundamentals of Conventional Linac Acceptance Testing**

Conventional Linear Accelerators (linac) are the major workforce machines in radiation oncology. The functionalities and performances of linac are set at the time of initial installation. Acceptance testing (AT) is important to check the satisfactory fulfill of the contractual specifications. Many technical details of linac performance at acceptance testing will be used as baselines for ongoing routine quality assurance. This session will discuss the technical specifications of the AT tests, and AT process and failure modes, and also the details included in the purchase contract. We will provide definition of performance specifications for major linac subsystems in acceptance testing procedures including imaging components, and special procedure components such as stereotactic radiosurgery (SRS) cones and micromultiple‐leaf collimator (MLC). We will make recommendations on the tests to be performed during the linac acceptance testing procedure. Also the emphasis would also be given on quantitative collection of relevant data during AT for periodic QA along several image‐based automated tests that are being performed during AT. Finally recommended responsibility of vendor in commissioning process would be discussed.

### Learning Objectives:

1. To learn the details of technical specifications in the AT test.
2. To understand the tests to be performed at acceptance testing and also AT process
3. To learn testing methods that complement vendor‐suggested measurements for beam matching and subsequent major repair/upgrades
4. To understand the automated tests and quantitative data that should be provided during AT for facilitating ongoing QA & upgraded.
5. Maximizing the overlap of AT and Commissioning process.

**Diagnostic Symposium ‐ SAM Grand Ballroom A**

***RDIM Software Development and Use***

MO‐D‐BRA‐00

## RDIM Software Development and Use

Moderator: Dustin Gress

*MD Anderson Cancer Center, Houston, TX*

MO‐D‐BRA‐01

J. Garrett

*Mississippi Baptist Medical Center*

**RDIM Software From the Ground Up: Demystifying RDIM**

This session will discuss the design strategy, inner workings, and clinical application of radiation dose index monitoring (RDIM) software. The session is intended to somewhat demystify RDIM products for attendees, inspire attendees to consider their own custom RDIM solutions, and to highlight opportunities for QA program enhancement using RDIM software.

**Learning Objectives:**

1. Understand design strategy or potential architecture of RDIM software;
2. Learn unique and little‐known resources to assist with RDIM software development; and
3. Appreciate opportunities for QA program enhancement at their own or their client institutions

Creator and owner of commercially available RDIM software product.

**TUESDAY, MARCH 8**

**Therapy Symposium ‐ SAM Grand Ballroom D**

***Commissioning & QA of Video‐Based Systems and Other Tools for Motion Management***

TU‐A‐BRD‐00

## Commissioning & QA of Video‐Based Systems and Other Tools for Motion Management

Moderator: Kyle Antes

*Presbyterian Healthcare System, Dallas, TX*

TU‐A‐BRD‐01

D. Shepard

*Swedish Cancer Institute*

**Choosing and Commissioning a Video‐Based Motion Management System**

TU‐A‐BRD‐02

H. Al‐Hallaq

*The University of Chicago*

**Clinical Implementation and On‐Going QA of a Video‐Based Motion Management System**

TU‐A‐BRD‐03

S. Hadley

*The University of Michigan*

**Clinical Use of Video‐Based Motion Management: Where Are We Now and Where Can We Go?**

A video‐based system can serve as an effective tool for both patient positioning and motion management. In this session, we will provide an overview of the commercially available solutions, as well as a comparison of their capabilities. We will discuss key considerations for selecting the right technology and the steps required in the commissioning process. Technical challenges will be described such as the impact of tissue deformations, reduced image quality for darker skin tones, nonspecific topography, inaccurate surface generation from the CT data, and the use of absolute versus relative positioning. The most common use of video systems is in breast radiotherapy. We will discuss coached deep inspiration breath‐hold (DIBH) using video monitoring and compare this to spirometry‐based techniques which require the patient to use a mouthpiece and nose clip. The role of video‐based systems for frameless stereotactic radiosurgery (SRS) and multiple target treatments will be presented, including design of immobilization for surface imaging of the head and patient monitoring to assure immobilization during delivery. The session will conclude with a discussion of the future potential applications of this technology.

**Learning Objectives:**

1. To understand the features of the different video‐based systems and how to choose and commission the system for your clinic.
2. To become familiar with clinical implementation of a video‐based system including breast, SRS, and deep inspiration breath‐hold applications.
3. To learn about the current state of video‐based systems, their technology and future applications.

**Diagnostic Symposium Grand Ballroom A**

***Medical Imaging Displays: Psychophysics and Quality Assurance***

TU‐A‐BRA‐00

## Medical Imaging Displays: Psychophysics and Quality Assurance

Moderator: Jeffrey Moirano

*University of Washington, Seattle, WA*

TU‐A‐BRA‐01

E. Krupinski

*Emory Univ*

**Psychophysics and the Human Visual System**

Display technology has changed dramatically since the inception of digital radiography and continues to change today as new technologies evolve and monitor characteristics improve. Deciding which monitor is the most appropriate for primary interpretation versus other reading tasks is critical but often confusing. Trying to decide what parameters are important to consider when purchasing a display and then how to maintain the optimal performance of those factors in a QA/QC program once purchased can be daunting.

The goal off this workshop is to provide an overview of modern displays for use in the clinical environment, examining performance expectations, QA/QC, and some basic aspects of the human visual system (psychophysics) that come into play when considering which displays should be used clinically.

The session will review the basics of available display types, performance and QA, expectations and requirements, clinical implementation, basic visual psychophysics as applied to display evaluation and image interpretation, and some of the ergonomic considerations involved in optimizing the reading room for viewing digital images.

**Learning Objectives:**

1. Understand the basics of medical image display technology
2. Learn what display parameters are important for clinical display QA/QC
3. Appreciate the role of the human visual system and its interaction with the display medium
4. Understand some of the basic ergonomic principles for healthful human‐computer interaction

**Diagnostic Symposium ‐ SAM Grand Ballroom A**

***Medical Imaging Displays: Psychophysics and Quality Assurance***

TU‐A‐BRA‐00

## Medical Imaging Displays: Psychophysics and Quality Assurance

Moderator: Jeffrey Moirano

*University of Washington, Seattle, WA*

TU‐A‐BRA‐01

M. Silosky

*University of Colorado School of Medicine*

**Display Monitor Performance and Evaluation**

In medical imaging, display performance has a direct impact on the information available to physicians for diagnoses. The duties of the imaging technologists also rely upon optimal display performance. Several accrediting bodies have implemented requirements to perform routine quality assurance for both primary and secondary displays. Professional organizations, including AAPM, have provided guidance regarding display QA, but changes in display technology require that these test procedures, and the expected performance metrics, be revisited.

In this session, the speaker will describe existing QA methodology for both primary and secondary displays, discuss limitations of these methods, and present some suggestions for revised QA procedures and performance metrics specific to the current display technology. He will also discuss the findings of a display QA program implemented in a large academic medical center.

**Learning Objectives:**

1. Understand the expectations and requirements for display QA from accrediting bodies
2. Review QA metrics, procedures, and guidance provided by AAPM
3. Identify some limitations of current QA procedures and opportunities for improving display QA

**Professional Symposium Grand Ballroom D**

***Medical Physics Practice Guidelines MPPG #7***

TU‐B‐BRD‐00

## Medical Physics Practice Guidelines MPPG #7

Moderator: Michael Howard

*Parkridge Medical Center, Chattanooga, TN*

TU‐B‐BRD‐01

J. Seibert

*UC Davis Medical Center*

**QMP Supervision of Medical Physicist Assistants — Imaging**

TU‐B‐BRD‐02

P. Halvorsen

*Lahey Clinic*

**QMP Supervision of Medical Physicist Assistants — Therapy**

## MEDICAL PHYSICS PRACTICE GUIDELINE: SUPERVISION OF MEDICAL PHYSICIST ASSISTANTS

Task Group #259 was assembled in March, 2014 under the Professional Council through the Practice Guidelines subcommittee of the AAPM. The committee charge is to develop a practice guideline to establish guidance for assigning medical physics related tasks and appropriate supervision levels for a Medical Physicist Assistant (MPA) under the direction of a Qualified Medical Physicist (QMP). A draft document has been completed and provides definitions, responsibilities of the QMP, responsibilities of the MPA, supervision plans, staffing/supervision ratios (MPA:QMP), and task‐related competency levels of the MPA verified by the QMP. A review of the document's content and the issues considered by the committee regarding therapy medical physics and diagnostic medical physics practices will be presented.

**Learning Objectives:**

1. Understanding of the reasoning and need behind TG 259
2. Understand the staff/supervision responsibilities
3. Understand the competency levels of the MPA

